# Passive decoy state quantum secure direct communication

**DOI:** 10.1016/j.fmre.2025.12.008

**Published:** 2025-12-27

**Authors:** Jia-Wei Ying, Jin-Yu Wang, Yu-Xiang Xiao, Shi-Pu Gu, Xing-Fu Wang, Lan Zhou, Yu-Bo Sheng

**Affiliations:** a College of Electronic and Optical Engineering and College of Flexible Electronics (Future Technology), Nanjing University of Posts and Telecommunications, Nanjing 210023, China; b School of Physics, Hangzhou Normal University, Hangzhou 311121, China; c College of Science, Nanjing University of Posts and Telecommunications, Nanjing 210023, China; d Institute of Quantum Information and Technology, Nanjing University of Posts and Telecommunications, Nanjing 210003, China

**Keywords:** Quantum communication, Quantum secure direct communication, Passive source, Decoy state, Single-photon-based

## Abstract

The decoy state method is an effective approach for addressing the source imperfections in quantum communication. By employing pulses of varying intensities to estimate error rates and yields, this method requires active modulation of the source intensity. However, the active modulation operation may introduce side-channel vulnerabilities, potentially allowing an eavesdropper to execute Trojan horse attacks. The passive decoy state method circumvents this issue by eliminating the need for active intensity modulation. In this paper, we propose a passive decoy state quantum secure direct communication (QSDC) protocol that generates weak coherent states with random intensities using two phase-randomized coherent pulses. Leveraging linear optics, our protocol facilitates straightforward implementation and is robust against eavesdropping side-channel attacks targeting source intensity modulators.We establish a system model for the protocol and derive its secrecy message capacity. Additionally, we optimize the input intensities of the protocol and obtain the maximum secrecy message capacity for each channel attenuation value. Compared with the previous protocol, our protocol exhibits an approximately 25% enhancement in the secrecy message transmission rate within a channel attenuation range of 6 dB. We also compare the performance of the three-decoy-state protocol with that of the infinite-decoy-state protocol. Within a channel attenuation range of 6.8 dB, the secrecy message capacity of the three-decoy-state protocol achieves approximately 94%–96% of that of the infinite-decoy-state.

## Graphical abstract

**Figure:**
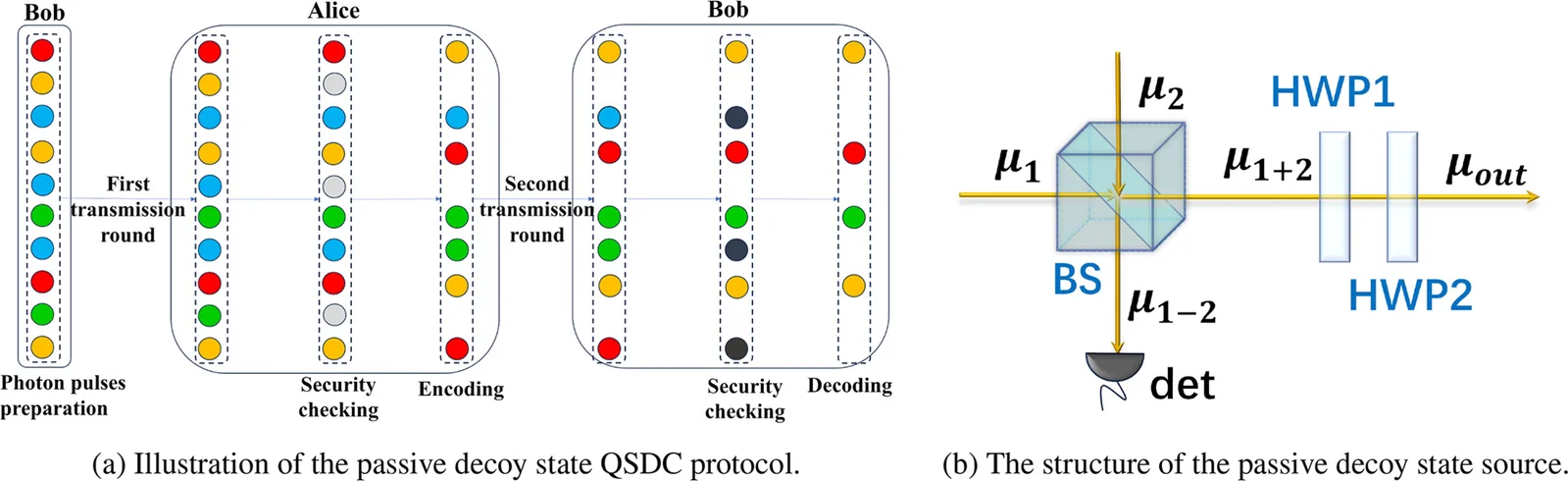

## 1 Introduction

Quantum communication has attracted considerable interest owing to its potential for providing unconditional security. Quantum key distribution (QKD) allows the secure key distribution between users. The first QKD protocol was proposed in 1984 [1]. In recent years, substantial theoretical [2], [3], [4], [5], [6], [7], [8], [9], [10], [11], [12], [13], [14], [15], [16], [17], [18] and experimental [19], [20], [21], [22], [23], [24], [25], [26], [27], [28], [29], [30], [31], [32] advancements have been made in QKD.

Quantum secure direct communication (QSDC) is a distinct branch of quantum communication that enables direct message transmission [33], [34], [35]. The concept of QSDC originated from Long and Liu [33] and was later refined in the entanglement-based QSDC [34], which utilizes the entangled photons and dense coding to transmit two bits of information per pulse. In 2004, a single-photon-based QSDC protocol was introduced [35]. In recent years, the theoretical framework of QSDC has experienced remarkable growth [36], [37], including Device-Independent (DI) protocols [38], [39], [40], Measurement-Device-Independent (MDI) protocols [41], [42], [43], [44], [45], one-step protocols [46], [47], [48], decoy state protocols [49], [50], [51], and high-dimensional protocols [52], [53], hybrid entanglement-based QSDC [54], among other important protocols [55], [56], [57], [58], [59], [60], [61]. On the experimental level, QSDC has also attained a wealth of achievements [62], [63], [64], [65], [66], [67], [68], [69], [70], [71], [72], such as single-photon-based QSDC [62], [63], entanglement-based QSDC [64], [65], QSDC network [66], [67], QSDC over 100-km fiber [68], and continuous variable QSDC [69], [70], [71], [72].

In theory, quantum communication offers unconditional security. However, inherent imperfections in actual devices can compromise this security [73], [74], [75], [76], [77]. Ideally, implementing a QKD protocol [1] requires a perfect single-photon source. In practice, commonly used sources generate weak coherent pulses (WCPs) that include vacuum states, single-photon states, and multi-photon states. In 2004, the Gottesman-Lo-Lütkenhaus-Preskill (GLLP) theory was proposed, providing a theoretical framework for analyzing quantum communication protocols using practical WCP sources for QKD [78]. Concurrently, the decoy state method was proposed and subsequently refined [79], [80], [81], enabling a more precise estimation of yields and error rates by generating multiple decoy state pulses with varying intensities. In recent years, the decoy state method has also been extended to QSDC [49], [50], [51].

The decoy state method effectively addresses source imperfections and mitigates risks posed by photon number splitting (PNS) attacks. Similar to QKD, single-photon-based decoy state QSDC [49], [50] require active modulation of the light pulse intensity, which can introduce new side-channel vulnerabilities. These vulnerabilities may allow eavesdroppers to execute Trojan horse attacks in QKD [82], [83], [84], [85], [86], [87], [88]. A promising solution to this issue is to utilize a passive source [89], [90], which eliminates the need for active modulation. Recently, the QSDC protocol based on the passive decoy state source was proposed [51]. However, it requires a parametric down-conversion (PDC) source like some passive decoy state QKD protocols [91], [92], [93], [94], [95], which infers the photon distribution in the signal path by measuring the photon number distribution of the idle light, resulting in a low secrecy message transmission rate. Interestingly, a passive decoy state method based on phase-randomized coherent pulses was proposed [90]. This method relies solely on linear optics and intensity detectors, facilitating straightforward implementation and has been applied in numerous quantum communication protocols [96], [97], [98], [99], [100], [101], [102], [103], [104].

Inspired by previous work [90], [96], [97], we propose a passive decoy state QSDC protocol utilizing phase-randomized coherent pulses. Weak coherent states with random intensities can be generated from two phase-randomized coherent pulses. This protocol has several advantages. First, it does not rely on the active modulation of the decoy state and can be implemented using linear optics, thereby simplifying experimental operations. Second, it is designed to remove potential side-channel attacks from an eavesdropper targeting the light source modulator. Third, using WCPs, it achieves higher efficiency than the protocol using a PDC source. We model the protocol and derive its secrecy message capacity. To achieve optimal performance, we optimize the intensity and provide the maximum secrecy message capacity that the protocol can achieve for each channel attenuation value, along with the corresponding input optical intensity. We also compare the performance of the three-decoy-state protocol with that of the infinite-decoy-state.

The structure of the paper is as follows. In Section 2, we introduce the source and protocol of passive decoy state QSDC. In Section 3, we give the system model of the passive decoy state QSDC and analyze the secrecy message capacity based on the decoy-state method. In Section 4, we conduct numerical simulation and parameter optimization. In Section 5, we give the discussion and conclusion.

## 2 The passive decoy state protocol

### 2.1 The passive decoy state source

In this section, we will first introduce the passive decoy state source. The structure of the passive decoy state source is shown in Fig. 1. Its input consists of two coherent pulses with identical intensity and random phases, which can be expressed as

(1) $$\begin{array}{c} |\mu_{1}\rangle =|\sqrt{\mu_{\mathrm{in}}}e^{i\phi_{1}}\rangle ,|\mu_{2}\rangle =|\sqrt{\mu_{\mathrm{in}}}e^{i\phi_{2}}\rangle , \end{array}$$

where $\mu_{in}$ is the intensity of two coherent pulses. $\phi_{1}$ and $\phi_{2}$ are random phases of two coherent pulses.

**Fig. 1:**
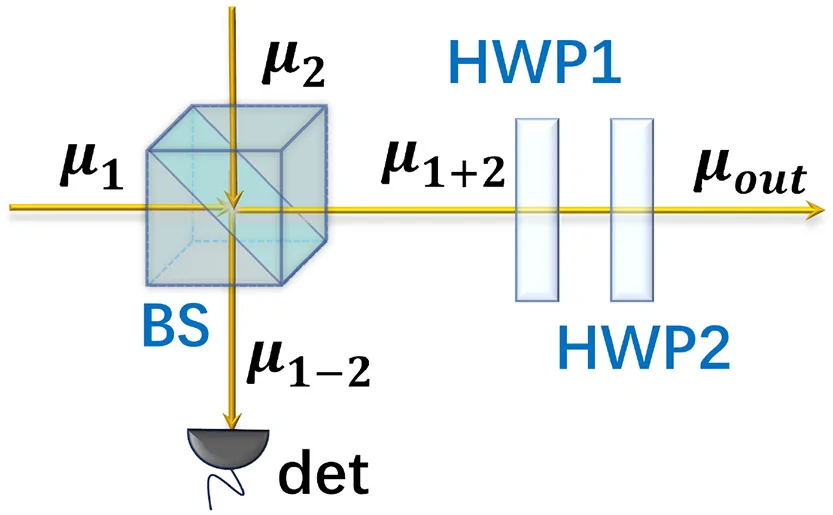
**The structure of the passive decoy state source, which is similar to that in**[90]. BS, t:(1−t) beam splitter; HWP1, $0^{\circ }$ half-wave plate when encode |H〉 or |+〉and $45^{\circ }$ half-wave plate when encode |V〉 or |−〉; HWP2, $0^{\circ }$ half-wave plate when encode |H〉 or |V〉 and $22.5^{\circ }$ half-wave plate when encode |+〉 or |−〉.

Then, the two input pulses undergo interference at a BS, generating outputs $|\mu_{1+2}\rangle$ and $|\mu_{1-2}\rangle$. This process can be deduced as follows

(2) $$\begin{array}{ccc} |\mu_{1}\rangle |\mu_{2}\rangle & = & |\sqrt{\mu_{\mathrm{in}}}e^{i\phi_{1}}\rangle |\sqrt{\mu_{\mathrm{in}}}e^{i\phi_{2}}\rangle \\ & & \overset{\mathrm{BS}}{\to }|\sqrt{2\mu_{\mathrm{in}}t}e^{i\frac{\phi_{1}+\phi_{2}}{2}}\cos\frac{\phi_{1}-\phi_{2}}{2}\rangle \\ & & \;|\sqrt{2\mu_{\mathrm{in}}\left(1-t\right)}e^{i\frac{\phi_{1}+\phi_{2}+\pi }{2}}\sin\frac{\phi_{1}-\phi_{2}}{2}\rangle \\ & = & |\mu_{1+2}\rangle |\mu_{1-2}\rangle , \end{array}$$

where t is the transmission coefficient of BS, and the detailed derivationis provided in Appendix C.

By measuring the intensity of pulse $|\mu_{1-2}\rangle$ as $2\mu_{in}\left(1-t\right)\sin^{2}\frac{\phi_{1}-\phi_{2}}{2}$, we can obtain the value of $\frac{\phi_{1}-\phi_{2}}{2}$. Let $\theta =\frac{\phi_{1}-\phi_{2}}{2}$, the intensity of $|\mu_{1+2}\rangle$ can be expressed as

(3) $$\begin{array}{c} \mu_{1+2}=\mu =2\mu_{in}t\cos^{2}\theta . \end{array}$$

According to the differences in pulse intensity $\mu_{1+2}$, the pulses $|\mu_{1+2}\rangle$ can be categorized into different groups.

Here, we classify the intensities into three groups, with two of them serving as decoy states and one as the signal state, and they are classified as follows

(4) $$\begin{array}{ccc} & & decoy\;state\;2=(0,\mu_{v}],\\ & & decoy\;state\;1=(\mu_{v},\mu_{d}],\\ & & signal\;state=(\mu_{d},\mu_{s}], \end{array}$$

where $\mu_{v},\mu_{d}$, and $\mu_{s}$ are the boundaries of three intensities. Here, we use ‘s’ to denote the signal, ‘d’ to denote the decoy, and ‘v’ to denote the vacuum, even though ‘decoystate2’ is not a genuine vacuum state. As shown in Fig. 2, when θ is within the interval ($\theta_{s1},\theta_{s2}$), the corresponding intensity range is ($\mu_{d},\mu_{s}$], and the emitted state is the signal state. When θ is within the intervals ($\theta_{d1},\theta_{s1}$] or [$\theta_{s2},\theta_{d2}$), the corresponding intensity range is ($\mu_{v},\mu_{d}$], and the emitted state is decoy state 1. When θ is within the intervals ($-\frac{\pi }{2},\theta_{d1}$] or [$\theta_{d2},\frac{\pi }{2}$), the corresponding intensity range is ($0,\mu_{v}$], and the emitted state is decoy state 2. Parameters $\theta_{d1}$, $\theta_{d2}$, $\theta_{s1}$, and $\theta_{s2}$ are selected based on practical requirements. Due to periodicity, when θ lies outside the interval ($-\frac{\pi }{2},\frac{\pi }{2}$), it is converted into this interval following a period of π.

**Fig. 2:**
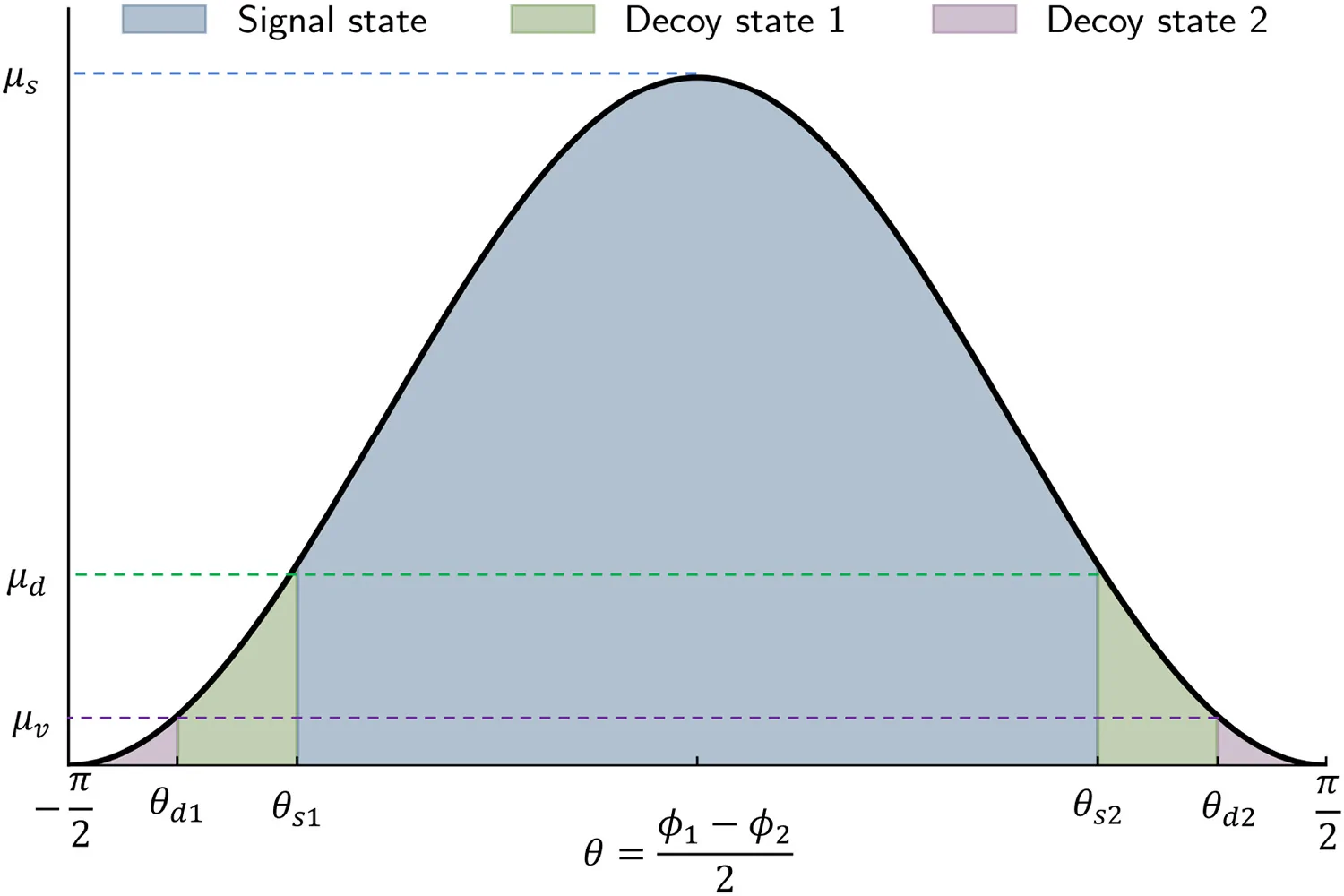
**The relationship between parameter**θ**and the classification of decoy state.**

Then, the pulse $|\mu_{1+2}\rangle$ will be fed into the encoder to produce the output state $|\mu_{out}\rangle$. The photons of $|\mu_{out}\rangle$ will be randomly encoded to |H〉, |V〉, |+〉, and |−〉. Here, |H〉 and |V〉 individually represent the horizontal and vertical polarization states of single photons, and they belong to the Z basis. $|\pm \rangle =\frac{1}{\sqrt{2}}(|H\rangle \pm \left|V\rangle \right)$ and they belong to the X basis.

### 2.2 The passive decoy state QSDC protocol

In this section, we introduce the passive decoy state QSDC protocol based on Ref. [35]. As shown in Fig. 3, the protocol involves two legitimate participants: the message sender Alice and the message receiver Bob. The detailed steps are described as follows.

**Fig. 3:**
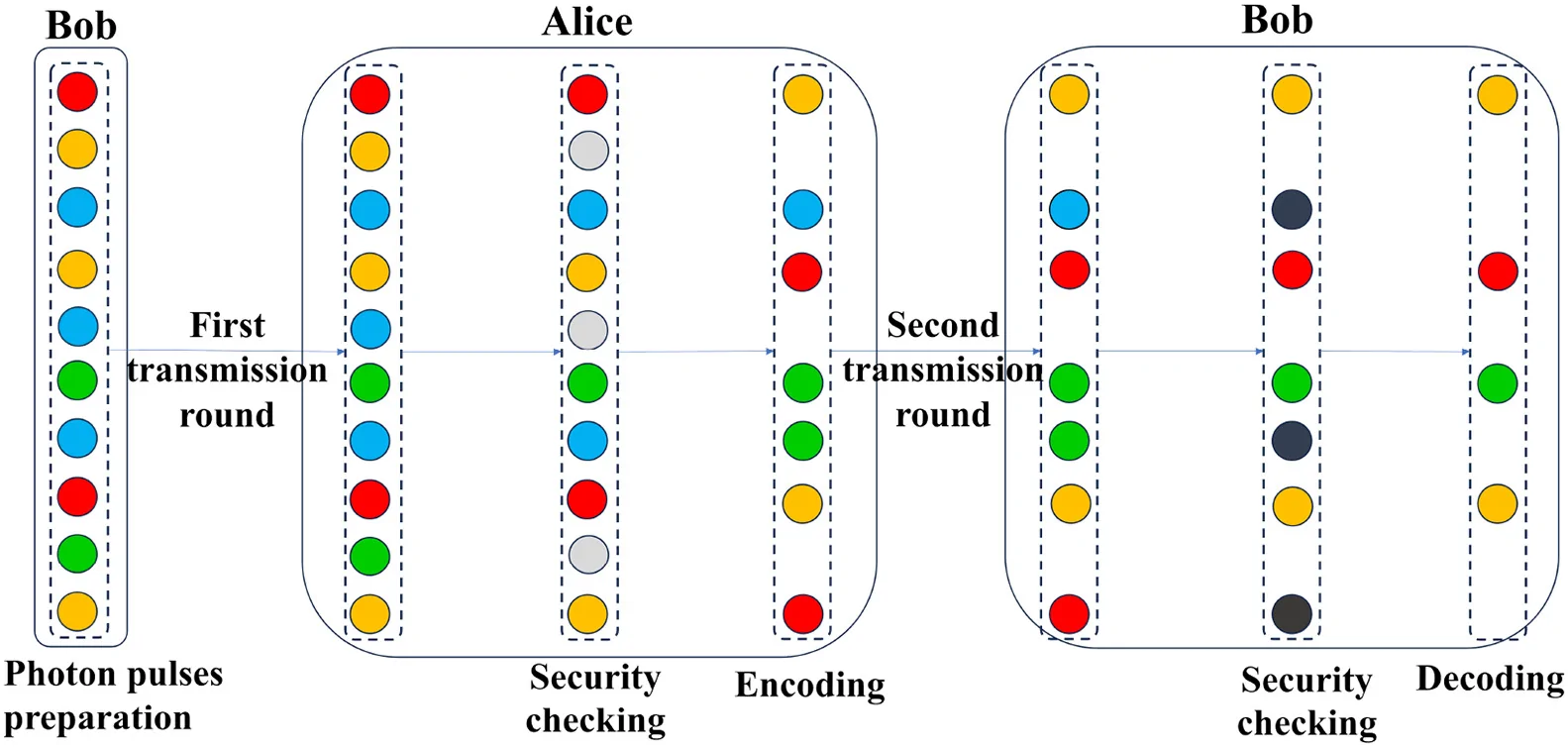
**Illustration of the passive decoy state QSDC protocol**. Each circle represents a photon pulse. The colors red, yellow, blue, and green denote the four kinds of encoded photons (X basis and Z basis). The gray circles indicate the pulses used in the first round of security checking, while the black circles represent the pulses utilized in the second round.

Step 1. Initial state preparation. Bob utilizes the passive decoy state source to generate a series of WCPs, denoted as sequence $S_{1}=\left\{S_{1}^{j}\right\}$, where $S_{1}^{j}$ represents the jth photon pulse in sequence $S_{1}$. As described in Section 2.1, these pulses have random decoy-state intensities and are randomly encoded as |H〉, |V〉, |+〉, and |−〉. Then Bob transmits these pulses to Alice.

Step 2. First round of security checking. After receiving these pulses, Alice first stores them and subsequently selects a subset for the first round of security checking, as the gray circles shown in Fig. 3, denoted as sequence $S_{2}=\left\{S_{2}^{j,k}\right\}\subseteq S_{1}$. Here, j represents the original position of the pulse in sequence $S_{1}$ and k represents the kth photon pulse in sequence $S_{2}$. Bob announces the information of the selected photons, including the initial encoding states and the decoy-state intensities. Alice performs decoy state analysis to estimate the error rate. If the error rate exceeds the threshold, communication is terminated; otherwise, it continues.

Step 3. Encoding. Alice selects another subset $S_{3}=\left\{S_{3}^{j,l}\right\}\subseteq S_{1}$ to be randomly encoded for the second round of security checking, as the black circles shown in Fig. 3, and encodes the message into the remaining photons as sequence $S_{4}=\left\{S_{4}^{j,m}\right\}\subseteq S_{1}$, where l(m) represents the l(m)th photon pulse in sequence $S_{3}(S_{4}$) and j represents the jth photon pulse in sequence $S_{1}$. Notice that $S_{2}\cup S_{3}\cup S_{4}=S_{1}$. The encoding operations can be expressed as $M_{0}=\sigma_{I}=\left|H\rangle \langle H\right|+\left|V\rangle \langle V\right|$ for the classical message 0 and $M_{1}=i\sigma_{Y}=\left|H\rangle \langle V\right|-\left|V\rangle \langle H\right|$ for 1. Then, Alice transmits the encoded photon pulse sequence to Bob.

Step 4. Second round of security checking. Bob decodes the received pulses based on the information of the initial encoding states. Then, Alice announces the labels (j and l) and encodes messages of the randomly encoded pulses in sequence $S_{3}$. Bob uses these messages for the second round of security checking to estimate the error rate. If the error rate exceeds the threshold, communication is terminated; otherwise, it continues.

Step 5. Message decoding. Bob restores the message sent by Alice through classical post-processing, utilizing the information from the other encoded photons in sequence $S_{4}$.

Here we present an example, as shown in Fig. 3. Suppose that the initial sequence is $S_{1}=\{|H\rangle$, |V〉, |+〉, |V〉, |+〉, |−〉, |+〉, |H〉, |−〉, |V〉}, corresponding to $S_{1}^{j},j\in \left[1,10\right]$. This sequence is then sent to Alice. Alice selects $S_{1}^{2}$, $S_{1}^{5}$ and $S_{1}^{9}$ to form the sequence $S_{2}=\left\{S_{2}^{2,1},S_{2}^{5,2},S_{2}^{9,3}\right\}$ for the first-round security checking. By measuring the photons in sequence $S_{2}$, an estimation of the bit error rate can be made. If the calculated bit error rate is lower than the threshold, the protocol advances to the next stage. Subsequently, the remaining photons are partitioned into two distinct sequences: $S_{3}=\left\{S_{3}^{3,1},S_{3}^{7,2},S_{3}^{10,3}\right\}$ and $S_{4}=\left\{S_{4}^{1,1},S_{4}^{4,2},S_{4}^{6,3},S_{4}^{8,4}\right\}$. Alice then randomly encodes the photons in sequence $S_{3}$, while encoding the sequence $S_{4}$ according to the messages she wants to transmit. For example, when aiming to transmit the binary sequence 1101, she can perform the operations $M_{1},M_{1},M_{0},M_{1}$ on pulses in $S_{4}$. As a result, the pulses in $S_{4}$ will transform to {|V〉, |H〉, |−〉, |V〉}. These encoded pulses are then sent to Bob. Upon receipt, Bob conducts measurements and carries out the second-round security checking. Once this security checking is passed, the messages sent by Alice can be deduced based on the initial state information ({|H〉, |V〉, |−〉, |H〉}). Through the execution of these steps, Bob is able to determine that the messages transmitted by Alice are 1101.

## 3 Secrecy message capacity and system model

### 3.1 The lower bound of the secrecy message capacity

The secrecy message capacity of single-photon-based QSDC under collective attack and PNS attack is given in Ref. [49], and shown as follows

(5) $$\begin{array}{ccc} C_{s} & = & \max_{\left\{p_{0}\right\}}\left\{I(A:B)-I(A:E)\right\}\\ & = & Q_{s}^{BAB}\left[1-h(E_{s}^{BAB})\right]-\{Q_{s,n=1}^{BAE}h\left(2e_{1}^{BA}\right)+\\ & & Q_{s,n=2}^{BAE}\left[\frac{1}{2}h\left(2e_{2}^{BA}\right)+\frac{1}{2}\right]+Q_{s,n\geq 3}^{BAE}\cdot 1\}. \end{array}$$

The subscript s means signal states. I(A:B) represents the mutual information between Alice and Bob, which is the classical information entropy that Bob can obtain by measuring the photons from Alice. It can be calculated by multiplying the overall gain of the second round of transmission ($Q_{s}^{BAB}$) by the entropy of a single photon pulse. The single-photon-based QSDC protocol [35] adopts two encoding operations, allowing for the transmission of 1 bit of information per pulse. The entropy of a single photon pulse is calculated as the theoretical transmission entropy minus the entropy of theerror rate in the second round of transmission $\left(E_{s}^{BAB}\right)$, which can be calculated with the binary Shannon entropy function h(x), and $h\left(x\right)=-x\log_{2}\left(x\right)-\left(1-x\right)\log_{2}\left(1-x\right)$. I(A:E) denotes the mutual information between Alice and Eve, which is the information entropy that Eve can steal via attacks on Alices encoded photons. In Appendix A, we mainly consider the collective attack and PNS attack to bound I(A:E) according to the von Neumann entropy. $p_{0}$ is the probability that Alice applies the encoding operation $M_{0}$ during her information encoding. In the context of PNS attacks and collective attacks, the mutual information I(A:E) stolen by Eve consists of three components. Eve can extract $h(2e_{1}^{BA})$ information from single-photon errors through collective attacks. For the two-photon case, Eve can first perform PNS attacks to capture one photon, then conduct collective attacks on the remaining photon to access $\frac{1}{2}h\left(2e_{2}^{BA}\right)+\frac{1}{2}$ information. When the number of photons exceeds two, Eve can perform unambiguous discrimination [105] and obtain all the information (1 bit) contained in the photon pulse. Where $e_{1}^{BA}$ ($e_{2}^{BA}$) is the single-photon (two-photon) error rate of the first-round transmission. $Q_{s,n=1}^{BAE}$, $Q_{s,n=2}^{BAE}$, and $Q_{s,n\geq 3}^{BAE}$ represent the gains associated with different photon numbers intercepted by Eve during the first-round transmission, shown as follows [49]

(6) $$\begin{array}{ccc} Q_{s}^{BAE} & = & \sum_{n=0}^{\infty }Q_{s,n}^{BAE}\\ & \leq & \sum_{n=0}^{\infty }\left[Q_{s,n}^{BA}-P\left(\mu ,n\right)Y_{0}^{A}\right]\max\left\{1,\frac{\gamma^{E}}{\gamma^{A}}\right\}, \end{array}$$

where $\gamma^{E}$ is the overall transmission efficiency of Eve after Alice encodes her receiving photons, which can be expressed as $t^{BA}\eta_{opt}^{E}\eta_{m}^{A}\eta_{opt}^{A}$. It includes the channel efficiency in the first-round transmission $t^{BA}$ and the optical device efficiency $\eta_{opt}^{E}$ introduced by Eve’s attack. Then, after memory and encoding by Alice, Eve can steal the information of the photons emitted by Alice. $\eta_{m}^{A}$ is the memory efficiency of Alice, and $\eta_{opt}^{A}$ is the optical device efficiency caused by Alice’s encoding operation. And $\gamma^{A}$ is the overall transmission efficiency for photons received and then measured by Alice, which can be defined as $t^{BA}\eta_{D}^{A}$. It includes the channel efficiency in the first-round transmission $t^{BA}$ and the detection efficiency $\eta_{D}^{A}$ of Alice. In the following analysis, we assume that $\eta_{opt}^{E}\eta_{m}^{A}\eta_{opt}^{A}$ is less than $\eta_{D}^{A}$. $Q_{s,n}^{BA}$ is the overall gain of n photons during the first-round transmission. P(μ,n) is the photon-number probability distribution. $Y_{0}^{A}$ is the zero-photon yield at Alice in the first-round transmission. More details in Appendix A.

### 3.2 The system model

In this section, we establish a system model to estimate the theoretical secrecy message capacity, including the yield, error rate, overall gain, and quantum bit error rate (QBER). The security of the protocol does not depend on this model. The model is only used to analyze the theoretical performance of the protocol. The n-photon yield $Y_{n}^{par}$ can be expressed as [80], [81]

(7) $$\begin{array}{c} Y_{n}^{par}=1-\left(1-Y_{0}^{par}\right){\left(1-\eta^{chan}\right)}^{n}. \end{array}$$

The item $\left(1-Y_{0}^{par}\right){\left(1-\eta^{chan}\right)}^{n}$ means that no zero-photon response occurs, and all n photons in the pulse are lost. Thus, $1-\left(1-Y_{0}^{par}\right){\left(1-\eta^{chan}\right)}^{n}$ represents that the source emits n photons and the detector responds. Where $Y_{0}^{par}$ is the zero-photon yield, and in the single-photon-based QSDC protocol, it is equal to 2Pd(1−Pd). Pd is the dark count probability. The superscript chan represents the two rounds of transmission. We denote the first round from Bob to Alice as BA, and the second round from Alice to Bob as BAB and chan∈{BA,BAB}. The superscript par represents the two participants Alice and Bob, and par∈{A,B}. $\eta^{chan}$is the transmission efficiency and can be expressed as follows

(8) $$\begin{array}{cc} \eta^{chan} & =t^{chan}\eta_{opt}^{par}\eta_{m}^{par}\eta_{D}^{par}, \end{array}$$

where $t^{chan}$ is the channel transmission efficiency, $\eta_{opt}^{par}$ is the intrinsic optical efficiency of the devices, $\eta_{m}^{par}$ is the efficiency of the quantum memory, and $\eta_{D}^{par}$ is the efficiency of detectors.

Considering the fiber loss channels, $t^{chan}$ can be further written as

(9) $$\begin{array}{c} t^{chan}=10^{-\frac{\alpha^{chan}}{10}}, \end{array}$$

where $\alpha^{chan}$ is the quantum channel attenuation corresponding to the transmission round.

The error rate is given by Lo et al. [80], Ma et al. [81]

(10) $$\begin{array}{c} e_{n}^{par}=\frac{e_{0}^{par}Y_{0}^{par}+e_{d}^{par}\left(1-{\left(1-\eta^{chan}\right)}^{n}\right)}{Y_{n}^{par}}. \end{array}$$

The error rate consists of two components: the zero-photon response error and the n-photon response error. Among them, the zero-photon response may be either correct or incorrect, thus $e_{0}^{par}$ is set to 0.5. And $e_{d}^{par}$ represents the detector error rate induced by basis mismatch in the detector response.

The overall gain is expressed as

(11) $$\begin{array}{c} Q_{S}^{chan}=\sum_{n=0}^{\infty }P_{S}\left(n\right)Y_{n}^{par}, \end{array}$$

where S∈{decoystate1,decoystate2,signalstate}, and $P_{S}\left(n\right)$ is the probability that the source emits n photons. Since the intensity we choose is an interval, we need to integrate the interval, and

(12) $$\begin{array}{ccc} P_{S}\left(n\right) & = & \frac{1}{P_{S}}\int_{S}\frac{\mu^{n}e^{-\mu }}{n!}f\left(\mu \right)d\mu ,\\ P_{S} & = & \int_{S}f\left(\mu \right)d\mu , \end{array}$$

where f(μ) is the probability density function and $P_{S}$ is the normalized probability. According to Eq. (2), the intensity $\mu =2\mu t\cos^{2}\frac{\phi_{1}-\phi_{2}}{2}$ is related to the random phases $\phi_{1}$ and $\phi_{2}$. Here, we assume that $\phi_{1}$ and $\phi_{2}$ are uniformly distributed. Let $\theta =\frac{\phi_{1}-\phi_{2}}{2}$, and owing to the periodicity of the cosine function, θ can be equivalently considered to follow a uniform distribution within its domain with respect to μ. According to the transformation formula for random variables, we have

(13) $$\begin{array}{c} f\left(\mu \right)=\frac{f(\theta )}{\left|J\right|}, \end{array}$$

where |J| is Jacobian determinant, and

(14) $$\begin{array}{c} \left|J\right|=\left|\frac{d\mu }{d\theta }\right|=4\mu_{in}t\cos\theta \sin\theta . \end{array}$$

Thus,

(15) $$\begin{array}{ccc} f(\mu ) & = & \frac{f(\theta )}{\left|J\right|}=\frac{1}{\Delta \theta }\frac{1}{4\mu_{in}t\cos\theta \sin\theta }\\ & = & \frac{1}{\Delta \theta }\frac{1}{2\sqrt{\mu }\sqrt{2\mu_{in}t-\mu }}, \end{array}$$

where Δθ is the domain of θ. According to the normalization conditions of the probability density function, we have

(16) $$\begin{array}{ccc} \int_{0}^{2\mu t}f\left(\mu \right)d\mu & = & 1\Rightarrow \Delta \theta =\frac{\pi }{2},\\ f(\mu ) & = & \frac{1}{\pi }\frac{1}{\sqrt{\mu }\sqrt{2\mu_{in}t-\mu }}. \end{array}$$

The overall gain can be further calculated as

(17) $$\begin{array}{ccc} Q_{S}^{chan} & = & \sum_{n=0}^{\infty }P_{S}\left(n\right)Y_{n}^{par}\\ & = & \frac{1}{P_{S}}\int_{S}\left(1-\left(1-Y_{0}^{par}\right)e^{-\mu \eta^{chan}}\right)f\left(\mu \right)d\mu . \end{array}$$

The QBER can be calculated as

(18) $$Q_{S}^{chan}E_{S}^{chan}=\sum_{n=0}^{\infty }P_{S}\left(n\right)e_{n}^{par}Y_{n}^{par}$$

(19) $$\begin{array}{ccc} & & =\frac{1}{P_{S}}\int_{S}\left(e_{0}^{par}Y_{0}^{par}+e_{d}^{par}-e_{d}^{par}e^{-\mu \eta^{chan}}\right)f\left(\mu \right)d\mu ,\\ & & E_{S}^{chan}=\frac{1}{P_{S}Q_{S}^{chan}}\int_{S}\left(e_{0}^{par}Y_{0}^{par}+e_{d}^{par}-e_{d}^{par}e^{-\mu \eta^{chan}}\right)f\left(\mu \right)d\mu . \end{array}$$

The details can be seen in Appendix D

### 3.3 Decoy state method

To estimate the secrecy message capacity, it is also necessary to evaluate the error rate and the yield. In this section, we use the decoy-state method for parameter estimation to estimate the error rates and yields of single-photon and two-photon states. In Ref. [11], the method for solving the upper bound of $e_{1}^{A}Y_{1}^{A}$ and the lower bound of $Y_{1}^{A}$ with linear programming are given by

(20) $$\begin{array}{ccc} \min & & Y_{1}^{A}\\ s.t. & & Q_{S}^{BA}\geq \sum_{n=0}^{n_{cut}}P_{S}\left(n\right)Y_{n}^{A},\\ & & Q_{S}^{BA}\leq \sum_{n=0}^{n_{cut}}P_{S}\left(n\right)Y_{n}^{A}+1-\sum_{n=0}^{n_{cut}}P_{S}\left(n\right),\\ & & 0\leq Y_{n}^{A}\leq 1, \end{array}$$

(21) $$\begin{array}{ccc} \max & & e_{1}^{A}Y_{1}^{A}\\ s.t. & & Q_{S}^{BA}E_{S}^{BA}\geq \sum_{n=0}^{n_{cut}}P_{S}\left(n\right)e_{n}^{A}Y_{n}^{A},\\ & & Q_{S}^{BA}E_{S}^{BA}\leq \sum_{n=0}^{n_{cut}}P_{S}\left(n\right)e_{n}^{A}Y_{n}^{A}+1-\sum_{n=0}^{n_{cut}}P_{S}\left(n\right),\\ & & 0\leq e_{n}^{A}Y_{n}^{A}\leq 1, \end{array}$$

and the estimated value of $e_{1}^{A}$ is

(22) $$\hat{e_{1}^{A}}=\frac{\hat{e_{1}^{A}Y_{1}^{A}}}{\hat{Y_{1}^{A}}},$$

where $\hat{Y_{1}^{A}}$ and $\hat{e_{1}^{A}Y_{1}^{A}}$ are the solutions of Eqs. (20) and (21), respectively.

We give the following linear programming problem to solve the upper bound of $e_{2}^{A}Y_{2}^{A}$ and the lower bound of $Y_{2}^{A}$ as

(23) $$\begin{array}{ccc} \min & & Y_{2}^{A}\\ s.t. & & P_{S1}\left(1\right)Q_{S2}^{BA}-P_{S2}\left(1\right)Q_{S1}^{BA}\geq \\ & & Y_{0}^{A}\left(P_{S2}\left(0\right)P_{S1}\left(1\right)-P_{S1}\left(0\right)P_{S2}\left(1\right)\right)\\ & & +\sum_{n=2}^{n_{cut}}Y_{n}^{A}\left(P_{S2}\left(n\right)P_{S1}\left(1\right)-P_{S1}\left(n\right)P_{S2}\left(1\right)\right),\\ & & P_{S1}\left(1\right)Q_{S2}^{BA}-P_{S2}\left(1\right)Q_{S1}^{BA}\leq \\ & & Y_{0}^{A}\left(P_{S2}\left(0\right)P_{S1}\left(1\right)-P_{S1}\left(0\right)P_{S2}\left(1\right)\right)\\ & & +\sum_{n=2}^{n_{cut}}Y_{n}^{A}\left(P_{S2}\left(n\right)P_{S1}\left(1\right)-P_{S1}\left(n\right)P_{S2}\left(1\right)\right)\\ & & +(P_{S1}\left(1\right)\left(1-\sum_{n=0}^{n_{cut}}P_{S2}\left(n\right)\right)\\ & & -P_{S2}\left(1\right)\left(1-\sum_{n=0}^{n_{cut}}P_{S1}\left(n\right))\right),\\ & & 0\leq Y_{n}^{A}\leq 1, \end{array}$$

(24) $$\begin{array}{ccc} \max & & e_{2}^{A}Y_{2}^{A}\\ s.t. & & P_{S1}\left(1\right)E_{S2}^{BA}Q_{S2}^{BA}-P_{S2}\left(1\right)E_{S1}^{BA}Q_{S1}^{BA}\geq \\ & & e_{0}^{A}Y_{0}^{A}\left(P_{S2}\left(0\right)P_{S1}\left(1\right)-P_{S1}\left(0\right)P_{S2}\left(1\right)\right)\\ & & +\sum_{n=2}^{n_{cut}}e_{n}^{A}Y_{n}^{A}\left(P_{S2}\left(n\right)P_{S1}\left(1\right)-P_{S1}\left(n\right)P_{S2}\left(1\right)\right),\\ & & P_{S1}\left(1\right)E_{S2}^{BA}Q_{S2}^{BA}-P_{S2}\left(1\right)E_{S1}^{BA}Q_{S1}^{BA}\leq \\ & & e_{0}^{A}Y_{0}^{A}\left(P_{S2}\left(0\right)P_{S1}\left(1\right)-P_{S1}\left(0\right)P_{S2}\left(1\right)\right)\\ & & +\sum_{n=2}^{n_{cut}}e_{n}^{A}Y_{n}^{A}\left(P_{S2}\left(n\right)P_{S1}\left(1\right)-P_{S1}\left(n\right)P_{S2}\left(1\right)\right)\\ & & +\left[P_{S1}\left(1\right)\left(1-\sum_{n=0}^{n_{cut}}P_{S2}\left(n\right)\right)-P_{S2}\left(1\right)\left(1-\sum_{n=0}^{n_{cut}}P_{S1}\left(n\right)\right)\right],\\ & & 0\leq e_{n}^{A}Y_{n}^{A}\leq 1, \end{array}$$

where S1, S2 denote different intensity intervals in Eq. (4).

The estimated value of $e_{2}^{A}$ is

(25) $$\hat{e_{2}^{A}}=\frac{\hat{e_{2}^{A}Y_{2}^{A}}}{\hat{Y_{2}^{A}}},$$

where $\hat{Y_{2}^{A}}$ and $\hat{e_{2}^{A}Y_{2}^{A}}$ are the solutions of Eqs. (23) and (24), respectively.

## 4 Numerical simulation

In this section, we present numerical simulations for our passive decoy state QSDC protocol, and we optimize the intensity to find the optimal intensity that maximizes the secrecy message capacity at each channel attenuation. The parameters employed are from Ref. [49] and are detailed in Table 1.

**Table 1:** **Parameters used in the numerical simulation**[49].

$\eta_{opt}^{BA}$	$\eta_{opt}^{BAB}$	Pd	$e_{d}^{A}$	$e_{d}^{B}$	$\eta_{H(V)}$
0.21	0.088	$8\times 10^{-8}$	0.0131	0.0026	0.7

In Fig. 4, we show the variation curve of secrecy message capacity with channel attenuation for different intensities. The boundaries of the three intensity intervals we set are $\mu_{s}=\mu$, $\mu_{d}=0.1\mu$, and $\mu_{v}=0.05\mu$. The truncation value $n_{cut}$ for the decoy state method is set to 9. As illustrated in Fig. 4, the secrecy message capacity exhibits a decreasing trend at low channel attenuation as the intensity decreases. However, at high channel attenuation, the capacity initially increases before declining. This behavior is attributed to the influence of intensity on the probabilities of the source generating vacuum states, single-photon states, and multi-photon states. Specifically, as intensity diminishes, the likelihood of the source producing a vacuum state rises, resulting in a reduction in overall gain Q. Meanwhile, the probability of generating multi-photon components decreases, which in turn limits the amount of information that can be intercepted by the eavesdropper. This scenario creates a trade-off, highlighting the significance of selecting an appropriate intensity for optimizing protocol performance. To achieve this, we optimize the intensity to identify the optimal intensity for each channel attenuation. In Fig. 5, we give the maximum secrecy message capacity of the protocol under each channel attenuation (blue curve). The optimal intensities are shown in Fig. 6. It can be observed that as the channel attenuation increases, the optimal intensity of the protocol gradually decreases. This is primarily because under high attenuation conditions, the protocol becomes more sensitive to eavesdropping; furthermore, the gains brought by the two-photon component gradually diminish. This requires the light source pulses to contain fewer multi-photon components, which is reflected in the light source intensity as a decrease in the optimal intensity. When the channel attenuation is 2, 4, 6 dB (corresponding to communication distances of 5, 10, 15 km), the maximum secrecy message capacity is $1.33\times 10^{-3}$ bit/pulse (μ=0.253), $3.17\times 10^{-4}$ bit/pulse (μ=0.134), and $3.14\times 10^{-5}$ bit/pulse (μ=0.0477), respectively. Considering a typical optical fiber channel with an attenuation coefficient of 0.2 dB/km [106], the maximum communication distance of our protocol is 17.75 km (corresponding to a two-round transmission channel attenuation of 7.1 dB). We also compare the three decoy state protocol with the infinite decoy state protocol. Interestingly, as illustrated in Fig. 6, the optimal intensity for the infinite-decoy-state case is higher than that for the three-decoy-state case. This is primarily because the infinite decoy states enable more accurate estimation of the yield and error rate, thereby reducing eavesdropping opportunities for eavesdroppers. This allows the infinite-decoy-state protocol to achieve a larger secrecy message capacity by using a higher intensity. However, due to the trade-off between gain and eavesdropping—as illustrated in Fig. 5—the secrecy message capacities of the three-decoy state and infinite decoy state protocols remain quite close-even though there is a noticeable difference in their optimal intensities.

**Fig. 4:**
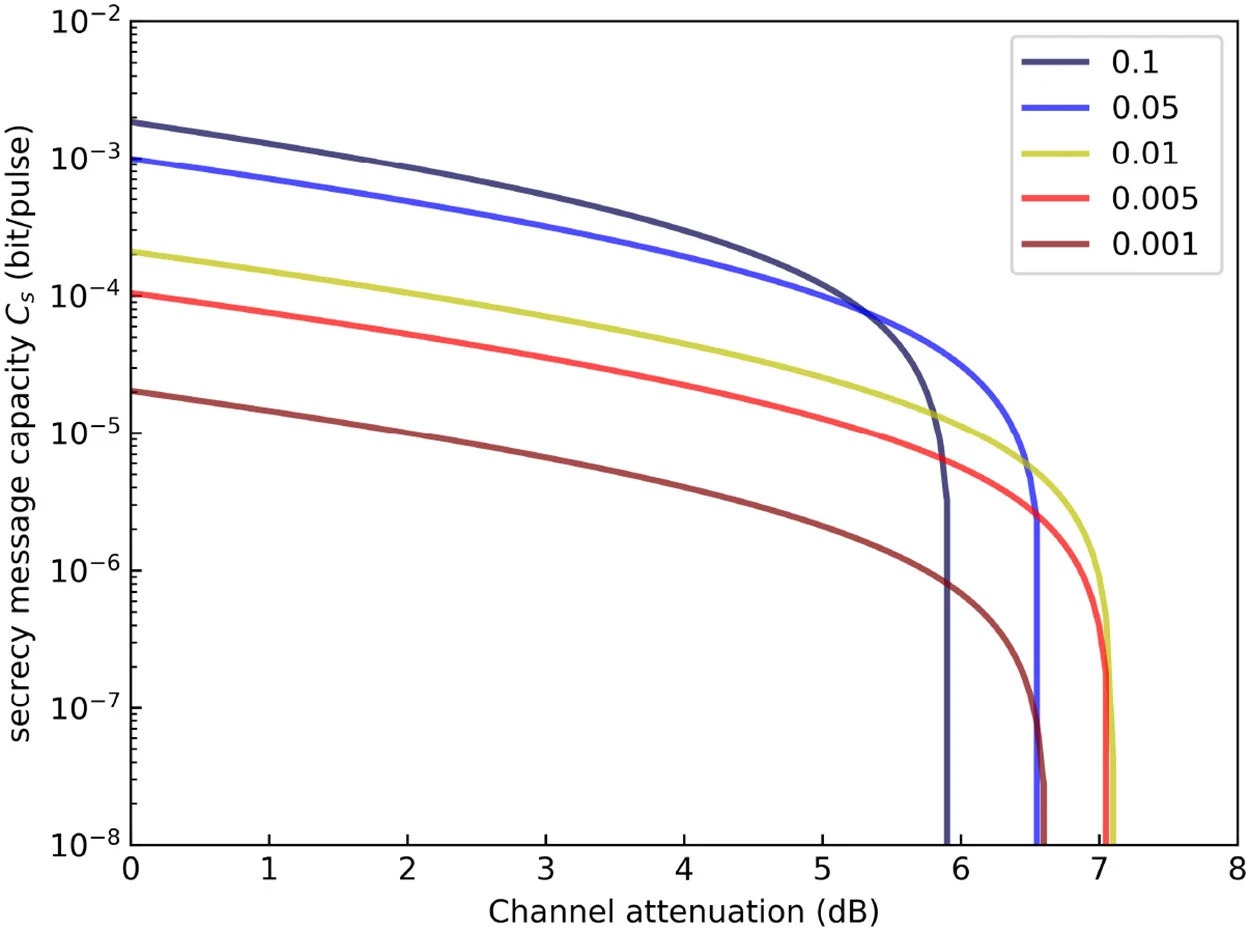
**Comparison of passive decoy state QSDC with different intensities**. The black, blue, yellow, red, and brown curves correspond to μ=0.1,0.05,0.01,0.005, and 0.001.

**Fig. 5:**
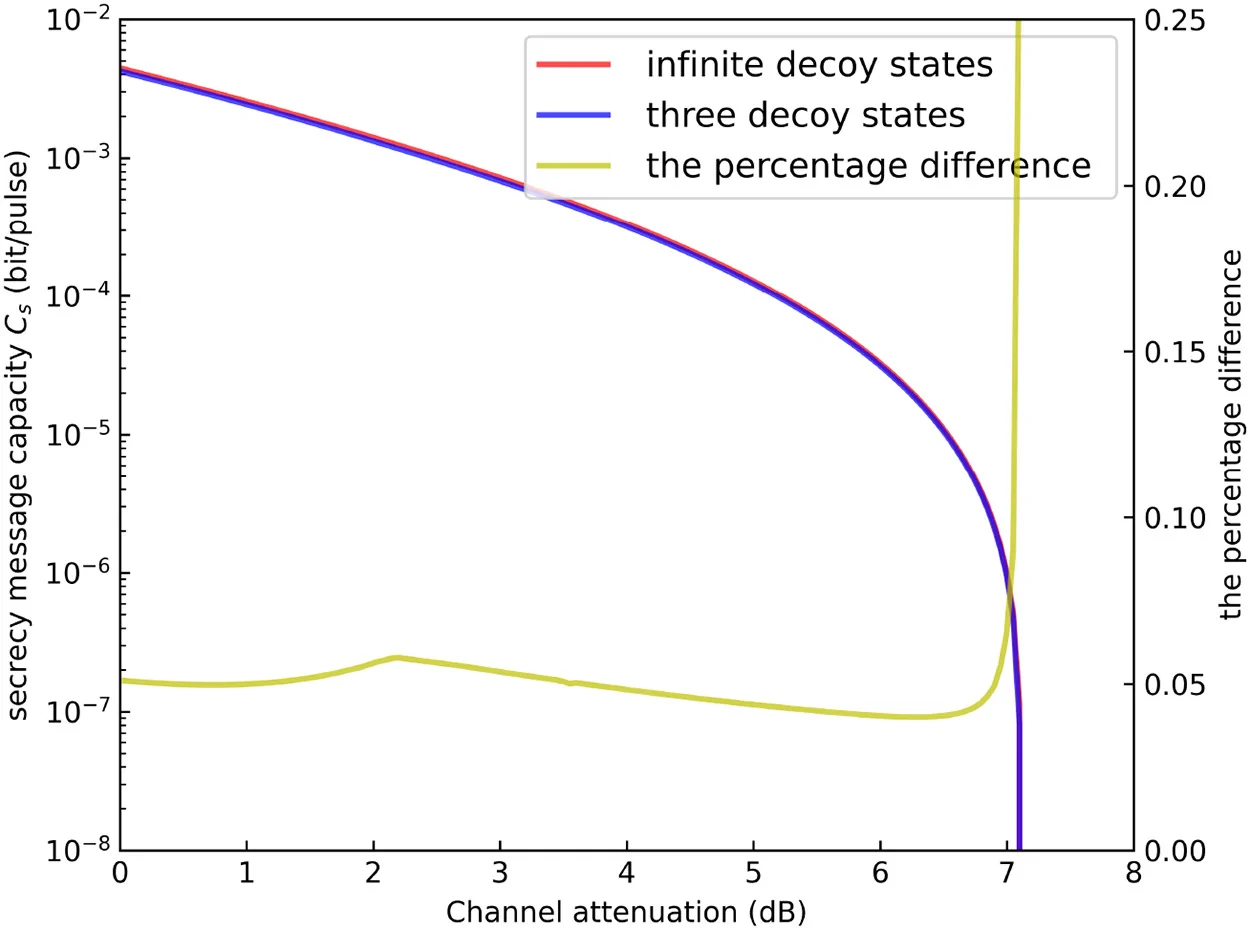
**The optimal secrecy message capacity of passive decoy state QSDC with three decoy states and infinite decoy states, and the percentage difference between them**. The left axis corresponds to the secrecy message capacity, while the right axis corresponds to the percentage difference. The red curve represents the maximum secrecy message capacity of three decoy states, and the blue curve represents the ideal protocol with infinite decoy states. The yellow curve represents the percentage difference between them.

In Fig. 5, we quantify their percentage difference(the yellow curve). We define the percentage difference, which is

(26) $$\begin{array}{c} r_{1}=\frac{C_{inf}-C_{tri}}{C_{inf}}, \end{array}$$

where $C_{inf}$ is the secrecy message capacity of the infinite-decoy-state protocol and $C_{tri}$ is that of the three-decoy-state protocol. The percentage difference demonstrates that the performance of the three-decoy-state is approximately 94%–96% of that of the infinite-decoy-state within 6.8 dB.

Meanwhile, we have further considered the influence of varying quantum memory in the protocol, as depicted in Fig. 7. The corresponding optimal intensity is shown in Fig. 8. Quantum memory efficiency can be regarded as equivalent to channel attenuation, and its principal effects on communication are manifested in the aspects of communication attenuation and the secrecy message capacity. Specifically, if the channel attenuation reaches 3 dB, as the memory efficiency drops from 1 to 0.9, the secrecy message capacity experiences a notable reduction of 44.14%. On the other hand, memory efficiency decreases from 0.9 to 0.8, and the secrecy message capacity also experiences another substantial decline of 56.36%.

**Fig. 6:**
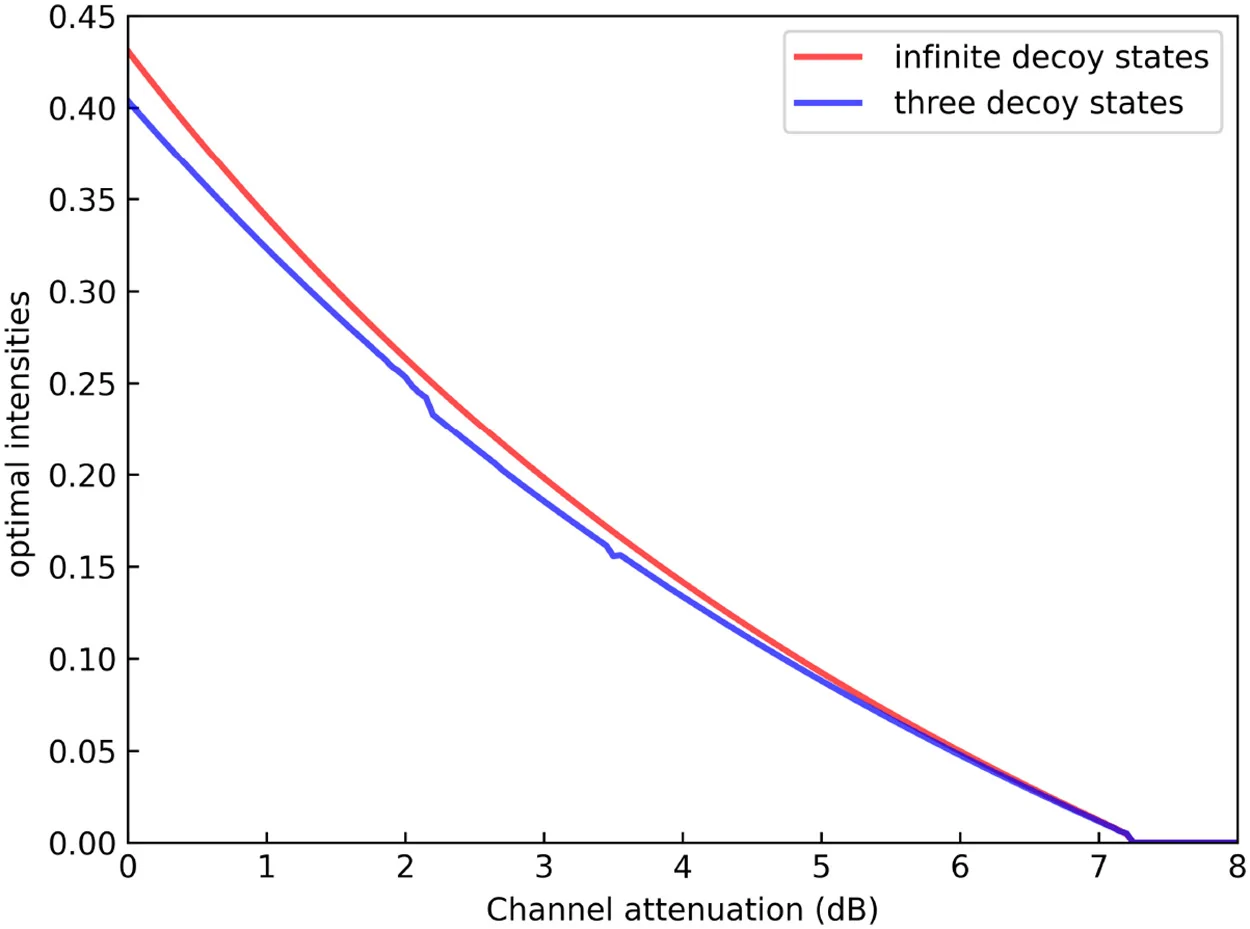
**The optimal intensity of passive decoy state QSDC**. The red and blue curves represent the optimal intensity of three decoy states and infinite decoy states, respectively.

**Fig. 7:**
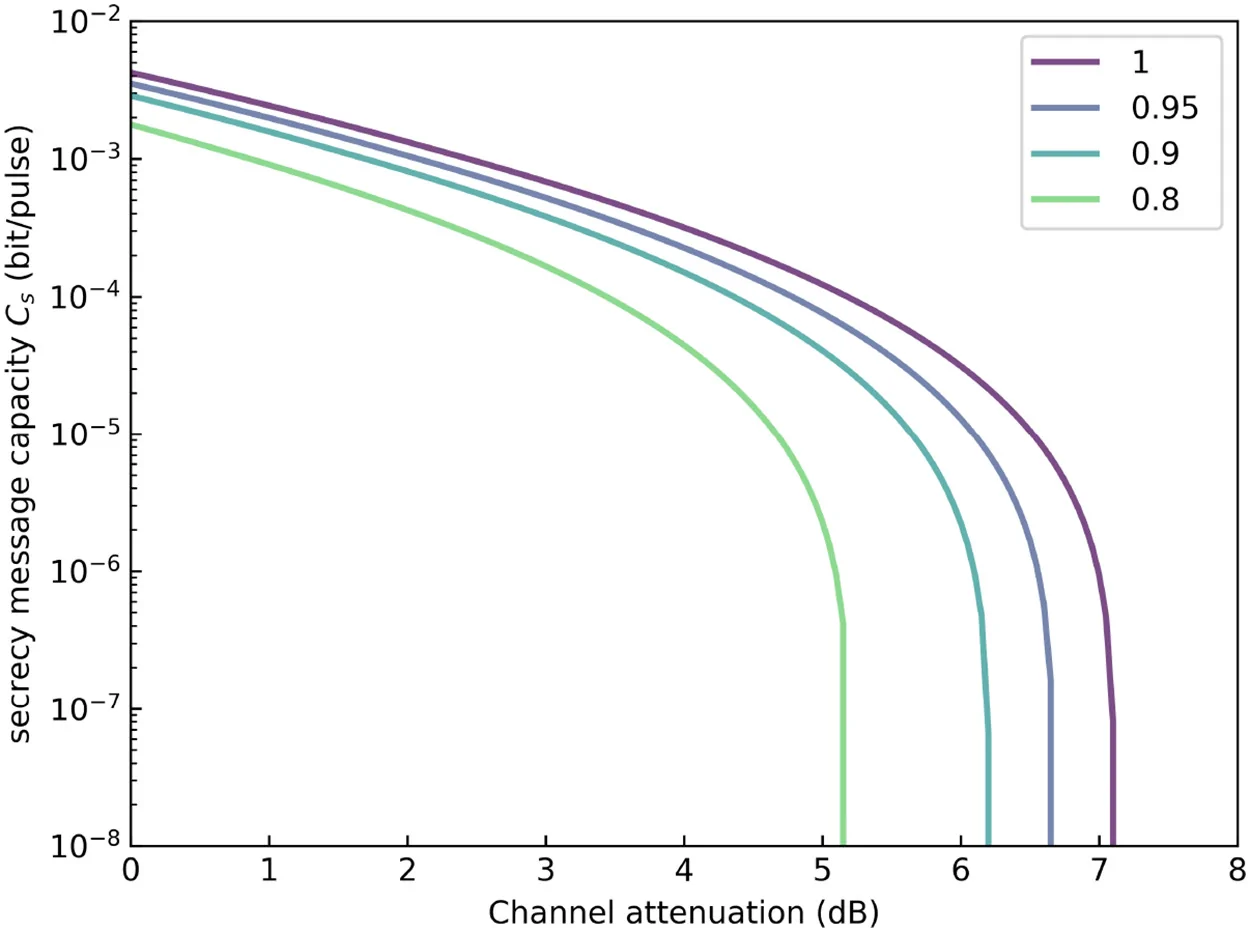
**The secrecy message capacity in relation to different memory efficiencies**. The curves in four distinct colors correspond respectively to the memory efficiencies of 1, 0.95, 0.9, and 0.8.

**Fig. 8:**
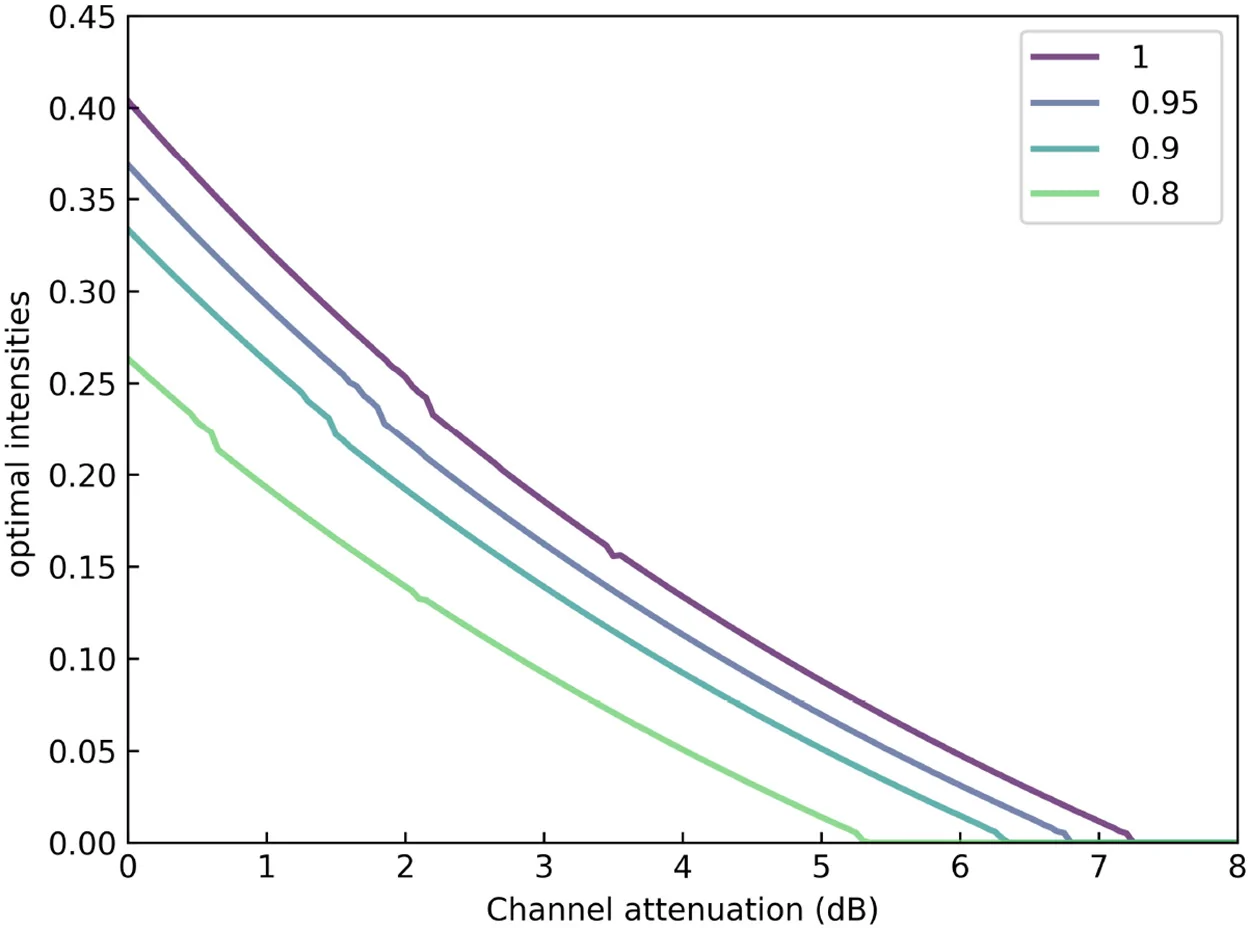
**The optimal intensity of the passive decoy state QSDC protocol in**Fig. 6. The curves in four distinct colors correspond respectively to the memory efficiencies of 1, 0.95, 0.9, and 0.8.

Furthermore, we have also compared the current research results with the previous work [51] and the single-photon-based QSDC protocol, with the results presented in Fig. 9. The red curve represents the protocol developed in this paper, the blue curve represents the protocol employing PDC, and the black curve represents the single-photon-based QSDC. It can be observed that the secrecy message transmission rates of both the passive protocol proposed in this paper and the PDC-based passive protocol are slightly lower than that of the actively modulated single-photon-based QSDC. Among them, the single-photon-based QSDC adopts coherent states following a Poisson distribution as the light source. The underlying reasons for this performance gap are distinct. For the protocol proposed in this paper, since coherent states are used to generate passive decoy states, the intensity of the output states is continuous within a specific interval. Such output states are equivalent to a mixture of states with different intensities, and the probability of n-photon components must be obtained by integrating over the entire intensity interval. Under these circumstances, the proportion of single-photon components in the photon number distribution of the resulting states is slightly lower than that incoherent states with a Poisson distribution, while the multi-photon components increase marginally. This leads to the light source quality being slightly inferior to that of coherent light sources, thereby reducing the secure information capacity and resulting in a maximum channel attenuation tolerance slightly lower than that of the single-photon-based QSDC. For the PDC-based protocol, although the single-photon component is significantly enhanced due to the heralding operation, numerous pulses are discarded due to heralding failures caused by factors such as detection efficiency and device loss. This leads to a reduction in resource utilization, which in turn degrades the secrecy message transmission rate. We have plotted the percentage difference in secrecy message transmission rate between this protocol and the one in Ref.[51]., which is shown as the yellow curve in Fig. 9. The percentage difference is defined as follows

(27) $$r_{2}=\frac{C_{this}-C_{ref}}{C_{ref}},$$

where $C_{this}$ represents the optimal secrecy message transmission rate of the protocol in this work and $C_{ref}$ is that of the protocol in Ref. [51]. The pulse frequency of coherent light sourceis set to 1 Hz (i.e., a unit pulse frequency). In the context of taking an ideal quantum memory into account, provided that the channel attenuation is roughly below 6 dB, its secrecy message transmission rate is around 25% higher than that of the preceding protocol.

**Fig. 9:**
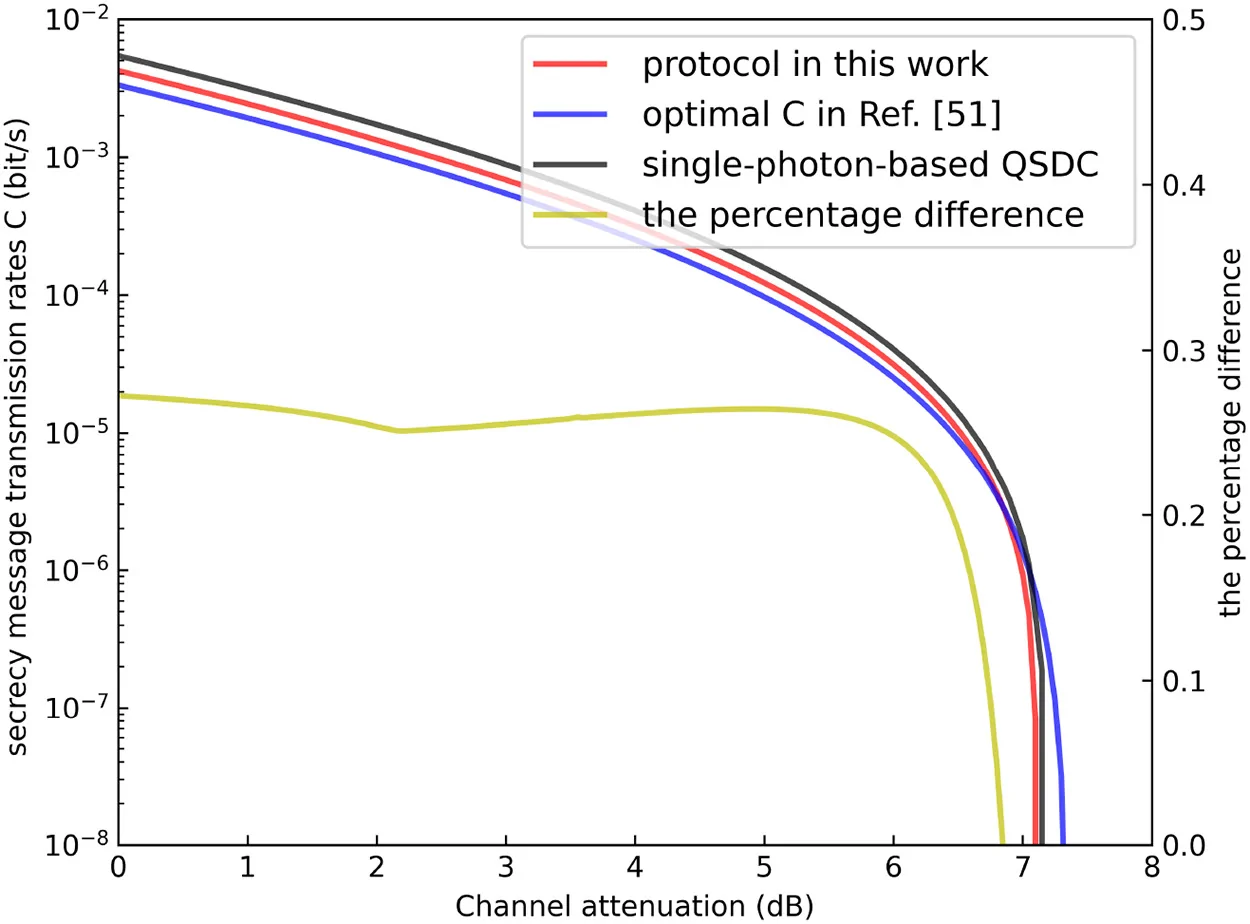
**The comparison of secrecy message transmission rate between this protocol and that in Ref.**[51]**and that of single-photon-based QSDC.** The left axis corresponds to the secrecy message transmission rate, while the right axis corresponds to the percentage difference. The red curve represents the protocol developed in this paper, the blue curve represents the protocol employing PDC, and the black curve represents the single-photon-based QSDC. The yellow curve represents the percentage difference in the secrecy message transmission rate between this protocol and the one in Ref. [51]. The pulse frequency of coherent light source is set to 1 Hz (i.e., a unit pulse frequency).

## 5 Discussion and conclusion

At present, the scenario considered in our protocol is relatively ideal, and several potential issues still need to be addressed during its practical implementation. First is the problem of phase randomization. Currently, we assume that the source can achieve perfect phase randomization, meaning its phase follows a uniform distribution within the range of 0 to 2π. However, in practical operations, this ideal state is not only costly but also difficult to truly achieve. A feasible alternative is to implement discrete phase randomization through active randomization [107], [108], [109], though this will add additional operational steps. It is worth noting that recent progress has been made in security analysis for laser sources with imperfect phase randomization [110], [111], which provides an important direction for the optimization of our protocol. Additionally, considering different noise scenarios [112]—such as the phase noise inherent to the system [113], [114]–is also a key aspect that requires focused attention in future research. Furthermore, our protocol is achieved under the infinite-length setting, which is an idealized hypothesis. Actually, in practical application, a finite block size can be realized. Due to statistical fluctuations, the secrecy message capacity of the protocol will experience a certain degree of degradation under the finite block size scenario. Recently, significant progress has been made in the analysis of the finite-length setting for QSDC based on preshared entanglement [56]. An important future research is to study the performance of the passive decoy state QSDC under the finite-length setting.

We have also analyzed the impact of memory efficiency on the protocol in the main text. Specifically, a reduction in memory efficiency will significantly compromise the protocol's secrecy message capacity. Currently, quantum memory technology has undergone experimental validation across multiple platforms [115], [116], [117], with breakthroughs achieved in both storage fidelity and efficiency [118], [119]—this lays a foundation for the practical implementation of memory-based QSDC. Meanwhile, several recent emerging protocols integrate QSDC with cryptography, successfully eliminating the reliance on memory [120], [121], [122]. Recently, a breakthrough experiment achieved an ultra-low QBER by utilizing time-bin and phase quantum states, extending the communication distance of QSDC to the 100 km scale [68]. They designed a quantum-memory-free (QMF) QSDC protocol with low-density Bose-Chaudhuri-Hocquenghem (LDBCH) codes, which eliminates the requirement for memory in QSDC-thereby significantly extending both the communication distance and secrecy message capacity of QSDC. This also offers valuable references for the practical deployment of our protocol.

In addition, achieving channel multiplexing via multicarrier orthogonal modulation technology [123], [124] is one of the effective means to improve the secrecy message capacity of QSDC. Its core lies in breaking through the single-channel transmission bottleneck through efficient division of spectrum resources. Meanwhile, the in-depth integration of QSDC technology with practical network services [125], [126] (such as remote sensing [127], etc.) is the key path to promote its transition from theory to practical application. It is worth noting that the cross-domain integration of quantum computing [128] and QSDC is more expected to further expand the performance boundaries and application scenarios of QSDC through the collaborative optimization of computing resources and communication links.

In conclusion, we propose a passive decoy state QSDC protocol. Two phase-randomized coherent pulses are interfered through a beam splitter to produce weakly coherent pulses with random intensity. Our protocol can be implemented with linear optics. By passively generating decoy states with varying intensities, we enhance resistance to side-channel attacks on source modulators. We model the protocol and estimate its secrecy message capacity using the decoy state method. Additionally, we optimize the input light intensity for each channel attenuation to maximize the secrecy message capacity. At the channel attenuation of 2, 4, 6 dB (corresponding to communication distances of 5, 10, 15 km), the maximum secrecy message capacity is $1.33\times 10^{-3}$ bit/pulse (μ=0.253), $3.17\times 10^{-4}$ bit/pulse (μ=0.134), and $3.14\times 10^{-5}$ bit/pulse (μ=0.0477), respectively. The maximum communication distance of the protocol is 17.75 km. On the other hand, the performance of the three-decoy-state protocol is compared with that of the infinite-decoy-state protocol. The performance of the three-decoy-state is approximately 94%-96% of that of the infinite-decoy-state. Furthermore, the influence of different memory efficiencies in the protocol are also taken into account. Ultimately, in comparison with the previous passive decoy-state protocol, our protocol in this paper exhibits a performance enhancement of around 25% when the channel attenuation is below 6 dB.

## CRediT authorship contribution statement

**Jia-Wei Ying:** Writing – original draft, Formal analysis, Data curation, Conceptualization. **Jin-Yu Wang:** Formal analysis, Data curation. **Yu-Xiang Xiao:** Software, Methodology. **Shi-Pu Gu:** Formal analysis, Data curation. **Xing-Fu Wang:** Software, Formal analysis. **Lan Zhou:** Writing – original draft, Supervision, Project administration, Funding acquisition. **Yu-Bo Sheng:** Supervision, Conceptualization.

## Declaration of competing interest

The authors declare that they have no conflicts of interest in this work.

## Appendix A The security proof of the single-photon-based QSDC protocol

In the main text, we use Eq. (5) to estimate the secrecy message capacity of our protocol. Its security proof has been provided in Ref. [49] and Ref. [63]. Here, we will briefly introduce the analysis process.

QSDC requires two rounds of transmission. In the first round, Bob transmits the initial state to Alice, and then Alice uses operations $M_{0}=\sigma_{I}=\left|H\rangle \langle H\right|+\left|V\rangle \langle V\right|$ and $M_{1}=i\sigma_{Y}=\left|H\rangle \langle V\right|-\left|V\rangle \langle H\right|$ for encoding. Since the quantum messages are encoded in the changes of the quantum states during the two rounds of transmission, the eavesdropper cannot obtain the encoded messages by only attacking the second round of transmission. Generally, the eavesdropper needs to launch attacks on both rounds of transmission. However, if the eavesdropper manages to obtain the information of the initial state in the first round of transmission, he can intercept all the photons in the second round for measurement. In this case, even if Eve’s attack can be detected, he has already acquired the encoded messages by comparing the quantum states of the two rounds of transmission. Therefore, in the analysis, we only consider the amount of information that Eve steals from the first round of transmission, assuming that he can obtain all the information about the states in the second round of transmission.

According to Wyner’s wiretap channel theory [129], the secrecy message capacity can be written as

(28) $$\begin{array}{cc} C_{s} & =\max_{\left\{p_{0}\right\}}\left\{I\left(A:B\right)-I\left(A:E\right)\right\}, \end{array}$$

where $p_{0}$ is the probability that Alice encodes message 0 with $M_{0}$ operation. According to Pan et al. [49] and Qi et al. [63], $p_{0}=\frac{1}{2}.\;I\left(A:B\right)$ is the mutual information between Alice and Bob, which represents the information entropy transmitted between Alice and Bob. Specifically, it can be expressed as the product of the gain of a single pulse and the amount of information carried by each pulse, and

(29) $$\begin{array}{ccc} I(A:B) & = & Q_{s}^{BAB}\left[h\left(p_{0}^{\prime }\right)-h\left(E_{s}^{BAB}\right)\right]\\ & = & Q_{s}^{BAB}\left[1-h(E_{s}^{BAB})\right], \end{array}$$

where $Q_{s}^{BAB}$ represents the overall gain of the signal state during the two rounds of transmission. $E_{s}^{BAB}$ represents the total error rate of the signal state during the two rounds of transmission. $h(p_{0}^{\prime })$ represents the amount of information entropy carried by each pulse after an error occurs. Among them, $p_{0}^{\prime }=p_{0}+E_{s}^{BAB}-2E_{s}^{BAB}p_{0}=p_{0}\left(1-E_{s}^{BAB}\right)+E_{s}^{BAB}\left(1-p_{0}\right)=\frac{1}{2}$ represents the probability of sending the message 0 after an error occurs, and $1-p_{0}^{\prime }$ represents the probability of sending the message 1. $h(E_{s}^{BAB})$ represents the information entropy lost due to errors. h(x) is the binary Shannon entropy function, given by $h\left(x\right)=-x\log_{2}\left(x\right)-\left(1-x\right)\log_{2}\left(1-x\right)$.

And I(A:E) is the mutual information between Alice and Eve, which represents the information entropy Eve can obtain from Alice. Here, we consider two types of attacks: the collective attack and the PNS attack. According to Pan et al. [49] and Qi et al. [63], the information entropy obtained by Eve can be decomposed into three components: that from single-photon states, that from two-photon states, and that from multi-photon states consisting of three or more photons. And I(A:E) can be expressed as

(30) $$\begin{array}{ccc} I(A:E) & = & Q_{s,n=1}^{BAE}I{\left(A:E\right)}_{n=1}\\ & & +Q_{s,n=2}^{BAE}I{\left(A:E\right)}_{n=2}+Q_{s,n\geq 3}^{BAE}I{\left(A:E\right)}_{n>2}, \end{array}$$

where $Q_{s,n=1}^{BAE}$, $Q_{s,n=2}^{BAE}$, and $Q_{s,n\geq 3}^{BAE}$ represent the gains associated with different photon numbers intercepted by Eve during the first round transmission, shown as Eq. (6).

For the case of single-photon states, Qi et al. derived the upper boundof information entropy that Eve can obtain in 2019 [63]. Firstly, Bob emits each of the four quantum states {|H〉, |V〉, |+〉, |−〉} with an equal probability of $\frac{1}{4}$. The single-photon states emitted by Bob can be written as the density matrix:

(31) $$\begin{array}{ccc} \rho_{1}^{B} & = & \frac{1}{4}\left(|H\rangle \langle H|+|V\rangle \langle V|+|+\rangle \langle +|+|-\rangle \langle -|\right)\\ & = & \frac{1}{2}\left(|H\rangle \langle H|+|V\rangle \langle V|\right). \end{array}$$

In the context of the collective attack, it is assumed that Eve applies the operation $U_{1}$ to execute a joint operation on Bob’s system and his own ancillary state |ε〉, and

(32) $$\rho_{1}^{BE}=U_{1}\left(\rho_{1}^{B}⊗\left|\varepsilon \rangle \langle \varepsilon \right|\right)U_{1}^{†},$$

where the effect of $U_{1}$ can be represented as [63]

(33) $$\begin{array}{ccc} U_{1}|H\rangle |\varepsilon \rangle & = & \left|H\rangle \right|\varepsilon_{00}\rangle +|V\rangle |\varepsilon_{01}\rangle =|\psi_{1}\rangle ,\\ U_{1}|V\rangle |\varepsilon \rangle & = & \left|H\rangle \right|\varepsilon_{10}\rangle +|V\rangle |\varepsilon_{11}\rangle =|\psi_{2}\rangle . \end{array}$$

After Alice performs the encoding, we have

(34) $$\begin{array}{ccc} M_{0}U_{1}|H\rangle |\varepsilon \rangle & = & \left|H\rangle \right|\varepsilon_{00}\rangle +|V\rangle |\varepsilon_{01}\rangle =|\psi_{1}\rangle ,\\ M_{0}U_{1}|V\rangle |\varepsilon \rangle & = & \left|H\rangle \right|\varepsilon_{10}\rangle +|V\rangle |\varepsilon_{11}\rangle =|\psi_{2}\rangle ,\\ M_{1}U_{1}|H\rangle |\varepsilon \rangle & = & \left|V\rangle \right|\varepsilon_{00}\rangle -|H\rangle |\varepsilon_{01}\rangle =|\psi_{3}\rangle ,\\ M_{1}U_{1}|V\rangle |\varepsilon \rangle & = & \left|V\rangle \right|\varepsilon_{10}\rangle -|H\rangle |\varepsilon_{11}\rangle =|\psi_{4}\rangle . \end{array}$$

Then the density matrix can be expressed as

(35) $$\begin{array}{ccc} \rho_{1}^{\mathrm{ABE}} & = & p_{0}M_{0}U_{1}\left(\rho_{1}^{B}⊗\left|\varepsilon \rangle \langle \varepsilon \right|\right)U_{1}^{†}M_{0}^{†}\\ & & +\;\left(1-p_{0}\right)M_{1}U_{1}\left(\rho_{1}^{B}⊗\left|\varepsilon \rangle \langle \varepsilon \right|\right)U_{1}^{†}M_{1}^{†}\\ & = & p_{0}\rho_{1}^{\mathrm{BE},0}+\left(1-p_{0}\right)\rho_{1}^{\mathrm{BE},1}\\ & = & p_{0}\frac{1}{2}\left(\left|\psi_{1}\rangle \langle \psi_{1}\left|+\right|\psi_{2}\rangle \langle \psi_{2}\right|\right)\\ & & +\;\left(1-p_{0}\right)\frac{1}{2}\left(\left|\psi_{3}\rangle \langle \psi_{3}\left|+\right|\psi_{4}\rangle \langle \psi_{4}\right|\right). \end{array}$$

According to Holevo bound [130], the upper bound of I(A:E) canbe expressed as

(36) $$I{\left(A:E\right)}_{n=1}\leq \max\left\{S\left(\rho_{1}^{ABE}\right)-p_{0}S\left(\rho_{1}^{BE,0}\right)-\;\left(1-p_{0}\right)S\left(\rho_{1}^{BE,1}\right)\right\},$$

where S(ρ) is von Neumann entropy, and $S\left(\rho \right)=-Tr\left(\rho \log_{2}\rho \right)=-\sum_{i}\lambda_{i}\log_{2}\lambda_{i}$. $\lambda_{i}$ are the eigenvalues of ρ. Since the operations performed by Alice and Eve are unitary operations, $S\left(\rho_{1}^{BE,0}\right)=S\left(\rho_{1}^{BE,1}\right)=S(\rho_{1}^{B}⊗|\varepsilon \rangle \langle \varepsilon |)=1$.

For $S(\rho_{1}^{ABE})$, since the non-zero eigenvalues of $\rho^{ABE}$ are identical to those of its corresponding Gram matrix [131], we can determine its von Neumann entropy by solving the Gram matrix. The element in the ith row and jth column of the Gram matrix can be defined as

(37) $$\begin{array}{c} G_{i,j}=\sqrt{p_{i}p_{j}}\langle \psi_{i}|\psi_{j}\rangle . \end{array}$$

By solving the eigenvalues of the Gram matrix, we are able to calculate$S(\rho_{1}^{ABE})$. When

(38) $$\begin{array}{ccc} & & \langle \varepsilon_{11}|\varepsilon_{10}\rangle =\langle \varepsilon_{00}|\varepsilon_{01}\rangle =0,\\ & & \langle \varepsilon_{10}|\varepsilon_{01}\rangle =-e_{Z},\\ & & \langle \varepsilon_{00}|\varepsilon_{11}\rangle =1-2e_{X}-2e_{Z}, \end{array}$$

$S(\rho_{1}^{ABE})$ reaches the maximum value as [63]

(39) $$S\left(\rho_{1}^{ABE}\right)=1+h\left(e_{Z}+e_{X}\right).$$

Assuming that the bit error and phase error of a single photon are equal to $e_{1}$,

(40) $$\begin{array}{c} I{\left(A:E\right)}_{n=1}=h\left(2e_{1}\right). \end{array}$$

For the two-photon states, the situation is similar. In 2020, Pan et al. demonstrated the upper bound of information entropy that Eve can obtain from two-photon states [49]. Bob emits each of the four quantum states {|HH〉, |VV〉, |++〉, |−−〉} with an equal probability of $\frac{1}{4}$. The two-photon states emitted by Bob can be written as the density matrix:

(41) $$\rho_{2}^{B}=\frac{1}{4}\left(|HH\rangle \langle HH|+|VV\rangle \langle VV|+|++\rangle \langle ++|+|--\rangle \langle --|\right).$$

The operations of Eve under PNS attack can be expressed as [49]

(42) $$\begin{array}{ccc} & & U_{2}{|HH\rangle }_{B}|\varepsilon \rangle ={|HH\rangle }_{B}|\varepsilon_{0000}{\rangle +|HV\rangle }_{B}|\varepsilon_{0001}\rangle =|\psi_{1}\rangle ,\\ & & U_{2}{|VV\rangle }_{B}|\varepsilon \rangle ={|VH\rangle }_{B}|\varepsilon_{1110}{\rangle +|VV\rangle }_{B}|\varepsilon_{1111}\rangle =|\psi_{2}\rangle ,\\ & & U_{2}{|+\rangle }_{B}{|+\rangle }_{B}|\varepsilon \rangle =|\psi_{3}\rangle ,\\ & & U_{2}{|-\rangle }_{B}{|-\rangle }_{B}|\varepsilon \rangle =|\psi_{4}\rangle , \end{array}$$

and after Alice perform $M_{1}$ operation, we have

(43) $$\begin{array}{ccc} & & M_{1}U_{2}{|HH\rangle }_{B}|\varepsilon \rangle ={|HV\rangle }_{B}|\varepsilon_{0000}{\rangle -|HH\rangle }_{B}|\varepsilon_{0001}\rangle =|\psi_{5}\rangle ,\\ & & M_{1}U_{2}{|VV\rangle }_{B}|\varepsilon \rangle ={|VV\rangle }_{B}|\varepsilon_{1110}{\rangle -|VH\rangle }_{B}|\varepsilon_{1111}\rangle =|\psi_{6}\rangle ,\\ & & M_{1}U_{2}{|++\rangle }_{B}|\varepsilon \rangle =|\psi_{7}\rangle ,\\ & & M_{1}U_{2}{|--\rangle }_{B}|\varepsilon \rangle =|\psi_{8}\rangle . \end{array}$$

After the operations of Eve and Alice, the initial state is transformed into

(44) $$\begin{array}{ccc} \rho_{2}^{ABE} & = & p_{0}\rho_{2}^{BE,0}+\left(1-p_{0}\right)\rho_{2}^{BE,1}\\ & = & p_{0}U_{2}(\rho_{2}^{B}⊗|\varepsilon \rangle \langle \varepsilon |)U_{2}^{†}\\ & & +\;\left(1-p_{0}\right)M_{1}U_{2}(\rho_{2}^{B}⊗|\varepsilon \rangle \langle \varepsilon |)U_{2}^{†}M_{1}^{†}\\ & = & \frac{p_{0}}{4}\left(|\psi_{1}\rangle \langle \psi_{1}\left|+\right|\psi_{2}\rangle \langle \psi_{2}\left|+\right|\psi_{3}\rangle \langle \psi_{3}\left|+\right|\psi_{4}\rangle \langle \psi_{4}|\right)\\ & & +\;\frac{\left(1-p_{0}\right)}{4}\left(|\psi_{5}\rangle \langle \psi_{5}\left|+\right|\psi_{6}\rangle \langle \psi_{6}\left|+\right|\psi_{7}\rangle \langle \psi_{7}\left|+\right|\psi_{8}\rangle \langle \psi_{8}|\right), \end{array}$$

where

(45) $$\rho_{2}^{BE,0}=\frac{1}{4}\left(|\psi_{1}\rangle \langle \psi_{1}\left|+\right|\psi_{2}\rangle \langle \psi_{2}\left|+\right|\psi_{3}\rangle \langle \psi_{3}\left|+\right|\psi_{4}\rangle \langle \psi_{4}|\right),$$

(46) $$\rho_{2}^{BE,1}=\frac{1}{4}\left(|\psi_{5}\rangle \langle \psi_{5}\left|+\right|\psi_{6}\rangle \langle \psi_{6}\left|+\right|\psi_{7}\rangle \langle \psi_{7}\left|+\right|\psi_{8}\rangle \langle \psi_{8}|\right).$$

According to the Holevo bound [130], the upper bound of $I{\left(A:E\right)}_{n=2}$ canbe expressed as

(47) $$I{\left(A:E\right)}_{n=2}\leq \max\left\{S\left(\rho_{2}^{ABE}\right)-p_{0}S\left(\rho_{2}^{BE,0}\right)-\left(1-p_{0}\right)S\left(\rho_{2}^{BE,1}\right)\right\},$$

where S(ρ) is von Neumann entropy, and $S\left(\rho \right)=-Tr\left(\rho \log_{2}\rho \right)=-\sum_{i}\lambda_{i}\log_{2}\lambda_{i}$. $\lambda_{i}$ are the eigenvalues of ρ. Since the operations performed by Alice and Eve are unitary operations, $S\left(\rho_{2}^{BE,0}\right)=S\left(\rho_{2}^{BE,1}\right)=S(\rho_{2}^{B}⊗|\varepsilon \rangle \langle \varepsilon |)=\frac{3}{2}$.

Similarly, we can calculate $S(\rho_{2}^{ABE})$ by solvingthe eigenvalues of the Gram matrix. Assuming that the bit error and phase error of two-photon states are equal to $e_{2}$, and when

(48) $$\begin{array}{ccc} & & \langle \varepsilon_{0000}|\varepsilon_{1110}\rangle =\langle \varepsilon_{0001}|\varepsilon_{1111}\rangle =0,\\ & & \langle \varepsilon_{0000}|\varepsilon_{0001}\rangle =\langle \varepsilon_{1110}|\varepsilon_{1111}\rangle =0,\\ & & \langle \varepsilon_{0001}|\varepsilon_{1110}\rangle =0,\\ & & \langle \varepsilon_{0000}|\varepsilon_{1111}\rangle =1-2e_{2}, \end{array}$$

$S(\rho_{2}^{ABE})$ reaches the maximum value as [49]

(49) $$\begin{array}{c} S\left(\rho_{1}^{ABE}\right)=2+\frac{1}{2}h\left(2e_{2}\right), \end{array}$$

and

(50) $$\begin{array}{c} I{\left(A:E\right)}_{n=2}=\frac{1}{2}+\frac{1}{2}h\left(2e_{2}\right). \end{array}$$

For the multi-photon states consisting of three or more photons, Eve can perform unambiguous discrimination [105] and obtain all the information (1 bit) contained in the photon pulse.

Thus, the secrecy message capacity can be expressed as [49]

(51) $$\begin{array}{ccc} C_{s} & = & Q_{s}^{BAB}\left[1-h\left(E_{s}^{BAB}\right)\right]-\{Q_{s,n=1}^{BAE}h\left(2e_{1}^{BA}\right)\\ & & +\;Q_{s,n=2}^{BAE}\left[\frac{1}{2}h\left(2e_{2}^{BA}\right)+\frac{1}{2}\right]+Q_{s,n\geq 3}^{BAE}\cdot 1\}. \end{array}$$

According to Eq. (2), in our protocol, the initial states generated by the source can be expressed as

(52) $$\rho_{1+2}=|\mu_{1+2}\rangle \langle \mu_{1+2}\left|=\right|\sqrt{\mu }e^{i\frac{\phi_{1}+\phi_{2}}{2}}〉〈\sqrt{\mu }e^{i\frac{\phi_{1}+\phi_{2}}{2}}|=\sum_{n=0}^{\infty }P\left(\mu ,n\right)|n\rangle \langle n|,$$

where P(μ,n) represents the probability that the source emits n photons, and $P\left(\mu ,n\right)=\frac{\mu^{n}e^{-\mu }}{n!}$.

After randomly encoding the initial states as |H〉, |V〉, |+〉, and |−〉,the density matrix becomes

(53) $$\begin{array}{ccc} \rho_{out} & = & \frac{1}{4}(|\mu_{1+2}\rangle_{a_{H}^{†}}\langle \mu_{1+2}\left|+\right|\mu_{1+2}\rangle_{a_{V}^{†}}\langle \mu_{1+2}|\\ & & +\;|\mu_{1+2}\rangle_{a_{+}^{†}}\langle \mu_{1+2}\left|+\right|\mu_{1+2}\rangle_{a_{-}^{†}}\langle \mu_{1+2}|). \end{array}$$

Since the intensities of the states emitted by Bob are randomly distributed within an interval, he groups the states whose intensities fall within the decoy-state interval. At this point, Bob emits a mixed state composed of coherent light with different intensities, and Bob’s state can be expressed as

(54) $$\begin{array}{ccc} \rho_{S} & = & \frac{1}{\int_{S}f\left(\mu \right)d\mu }\int_{S}\frac{1}{4}{(|\mu_{1+2}\rangle }_{a_{H}^{†}}\langle \mu_{1+2}\left|+\right|\mu_{1+2}\rangle_{a_{V}^{†}}\langle \mu_{1+2}|\\ & & +\;|\mu_{1+2}\rangle_{a_{+}^{†}}\langle \mu_{1+2}\left|+\right|\mu_{1+2}\rangle_{a_{-}^{†}}\langle \mu_{1+2}|)f\left(\mu \right)d\mu , \end{array}$$

where S represents the decoy-state interval and S∈{decoystate1,decoystate2,signalstate}.f(μ) is the probability density function, given in Eq. (16).

Among them, the n-photon states emitted by Bob can be expressed as

(55) $$\begin{array}{ccc} \rho_{S}^{n} & = & \frac{1}{\int_{S}P\left(\mu ,n\right)f\left(\mu \right)d\mu }\int_{S}\frac{P(\mu ,n)}{4}{(|n\rangle }_{a_{H}^{†}}\langle n|+{|n\rangle }_{a_{V}^{†}}\langle n|\\ & & +{\;|n\rangle }_{a_{+}^{†}}\langle n|+{|n\rangle }_{a_{-}^{†}}\langle n|)f\left(\mu \right)d\mu \\ & = & \frac{1}{4}\left({|n\rangle }_{a_{H}^{†}}\langle n|+{|n\rangle }_{a_{V}^{†}}\langle n|+{|n\rangle }_{a_{+}^{†}}\langle n|+{|n\rangle }_{a_{-}^{†}}\langle n|\right). \end{array}$$

It is easy to notice that the single-photon states and the two-photon states emitted by Bob are

(56) $$\begin{array}{cc} \rho_{S}^{1}= & \frac{1}{4}\left(|H\rangle \langle H|+|V\rangle \langle V|+|+\rangle \langle +|+|-\rangle \langle -|\right), \end{array}$$

(57) $$\rho_{S}^{2}=\frac{1}{4}\left(|HH\rangle \langle HH|+|VV\rangle \langle VV|+\;|++\rangle \langle ++|+|--\rangle \langle --|\right),$$

which are the same as Eqs. (31) and (41). Through derivation, we can obtain the same result as Eq. (51). Therefore, our protocol enables the utilization of Eq. (51) to analyze the secrecy message capacity of the protocol.

## Appendix B The yield $Y_{n}$ and error rate $e_{n}$

According to Eq. (52), after randomly encoding, the density matrix of the quantum state (taking (|H〉) as an example) can be written as

(58) $$\begin{array}{c} \rho_{H}=|\mu_{1+2}\rangle_{a_{H}^{†}}\langle \mu_{1+2}|=\sum_{n=0}^{\infty }{P\left(\mu ,n\right)|n\rangle }_{a_{H}^{†}}\langle n|. \end{array}$$

Since the intensity of the quantum states emitted by Bob is random, Bob classifies them into signal states, decoy states 1, and decoy states 2 based on post-selection intervals in Eq. (4). In this case, the density matrix of the output states needs to be obtained by integrating the states emitted by Bob over the corresponding intervals. Taking the signal states as an example, the density matrix of the signal states will be

(59) $$\begin{array}{ccc} \rho_{signal,H} & = & \frac{1}{\int_{\mu_{d}}^{\mu_{s}}f\left(\mu \right)d\mu }\int_{\mu_{d}}^{\mu_{s}}\sum_{n=0}^{\infty }{P\left(\mu ,n\right)|n\rangle }_{a_{H}^{†}}\langle n|f\left(\mu \right)d\mu \\ & = & \frac{1}{\int_{\mu_{d}}^{\mu_{s}}f\left(\mu \right)d\mu }\sum_{n=0}^{\infty }\int_{\mu_{d}}^{\mu_{s}}{P\left(\mu ,n\right)f\left(\mu \right)d\mu |n\rangle }_{a_{H}^{†}}\langle n|\\ & = & \sum_{n=0}^{\infty }P_{signal}{\left(n\right)|n\rangle }_{a_{H}^{†}}\langle n|, \end{array}$$

(60) $$\begin{array}{cc} P_{signal}\left(n\right)= & \frac{1}{\int_{\mu_{d}}^{\mu_{s}}f\left(\mu \right)d\mu }\int_{\mu_{d}}^{\mu_{s}}P\left(\mu ,n\right)f\left(\mu \right)d\mu , \end{array}$$

where $\frac{1}{\int_{\mu_{d}}^{\mu_{s}}f\left(\mu \right)d\mu }$ is the normalized coefficient. In this case, the source can be equivalent to generating an n-photon state with a probability of $P_{signal}\left(n\right)$. The n-photon yield $Y_{n}^{par}$ can be expressed as Eq. (7), which is the same as that in the traditional QKD protocol [80], [81] and is independent of the interval. Similarly, $e_{n}^{par}$ is also independent of the interval and can be expressed as Eq. (10).

## Appendix C The derivation of Eq. (2)

The two input pulses $|\mu_{1}\rangle$ and $|\mu_{2}\rangle$ undergo interference at a BS, generating outputs $|\mu_{1+2}\rangle$ and $|\mu_{1-2}\rangle$. This process can be deduced as

(61) $$\begin{array}{ccc} \left|\mu_{1}\rangle \right|\mu_{2}\rangle & = & |\sqrt{\mu_{in}}e^{i\phi_{1}}\rangle |\sqrt{\mu_{in}}e^{i\phi_{2}}〉\\ & & \overset{BS}{\to }|\frac{1}{\sqrt{2}}\left(\sqrt{\mu_{in}t}e^{i\phi_{1}}+\sqrt{\mu_{in}t}e^{i\phi_{2}}\right)〉\\ & & |\frac{1}{\sqrt{2}}\left(\sqrt{\mu_{in}\left(1-t\right)}e^{i\phi_{1}}-\sqrt{\mu_{in}\left(1-t\right)}e^{i\phi_{2}}\right)〉\\ & = & |\sqrt{\frac{\mu_{in}t}{2}}e^{i\frac{\phi_{1}+\phi_{2}}{2}}\left(e^{i\frac{\phi_{1}-\phi_{2}}{2}}+e^{-i\frac{\phi_{1}-\phi_{2}}{2}}\right)〉\\ & & |\sqrt{\frac{\mu_{in}\left(1-t\right)}{2}}e^{i\frac{\phi_{1}+\phi_{2}}{2}}\left(e^{i\frac{\phi_{1}-\phi_{2}}{2}}-e^{-i\frac{\phi_{1}-\phi_{2}}{2}}\right)〉\\ & = & |\sqrt{\frac{\mu_{in}t}{2}}e^{i\frac{\phi_{1}+\phi_{2}}{2}}2\cos\frac{\phi_{1}-\phi_{2}}{2}〉\\ & & |\sqrt{\frac{\mu_{in}\left(1-t\right)}{2}}e^{i\frac{\phi_{1}+\phi_{2}}{2}}2i\sin\frac{\phi_{1}-\phi_{2}}{2}〉\\ & = & |\sqrt{2\mu_{in}t}e^{i\frac{\phi_{1}+\phi_{2}}{2}}\cos\frac{\phi_{1}-\phi_{2}}{2}〉\\ & & |\sqrt{2\mu_{in}\left(1-t\right)}e^{i\frac{\phi_{1}+\phi_{2}+\pi }{2}}\sin\frac{\phi_{1}-\phi_{2}}{2}〉\\ & = & |\mu_{1+2}\rangle |\mu_{1--2}〉. \end{array}$$

## Appendix D The derivation of Eqs. (17) and (18)

The overall gain can be further calculated as

(62) $$\begin{array}{ccc} Q_{S}^{chan} & = & \sum_{n=0}^{\infty }P_{S}\left(n\right)Y_{n}^{par}\\ & = & \frac{1}{P_{S}}\sum_{n=0}^{\infty }Y_{n}^{par}\int_{S}\frac{\mu^{n}e^{-\mu }}{n!}f\left(\mu \right)d\mu \\ & = & \frac{1}{P_{S}}\int_{S}\sum_{n=0}^{\infty }Y_{n}^{par}\frac{\mu^{n}e^{-\mu }}{n!}f\left(\mu \right)d\mu \\ & = & \frac{1}{P_{S}}\int_{S}\sum_{n=0}^{\infty }\left(1-\left(1-Y_{0}^{par}\right){\left(1-\eta^{chan}\right)}^{n}\right)\frac{\mu^{n}e^{-\mu }}{n!}f\left(\mu \right)d\mu \\ & = & \frac{1}{P_{S}}\int_{S}\left(1-\left(1-Y_{0}^{par}\right)\sum_{n=0}^{\infty }\frac{{\left(\left(1-\eta^{chan}\right)\mu \right)}^{n}}{n!}e^{-\mu }\right)f\left(\mu \right)d\mu \\ & = & \frac{1}{P_{S}}\int_{S}\left(1-\left(1-Y_{0}^{par}\right)e^{-\mu \eta^{chan}}\right)f\left(\mu \right)d\mu . \end{array}$$

The QBER can be calculated as

(63) $$\begin{array}{cc} & Q_{S}^{chan}E_{S}^{chan}=\sum_{n=0}^{\infty }P_{S}\left(n\right)e_{n}^{par}Y_{n}^{par} \end{array}$$

(64) $$\begin{array}{ccc} & & =\frac{1}{P_{S}}\sum_{n=0}^{\infty }e_{n}^{par}Y_{n}^{par}\int_{S}\frac{\mu^{n}e^{-\mu }}{n!}f\left(\mu \right)d\mu \\ & & =\frac{1}{P_{S}}\int_{S}\sum_{n=0}^{\infty }e_{n}^{par}Y_{n}^{par}\frac{\mu^{n}e^{-\mu }}{n!}f\left(\mu \right)d\mu \\ & & =\frac{1}{P_{S}}\int_{S}\sum_{n=0}^{\infty }\left(e_{0}^{par}Y_{0}^{par}+e_{d}^{par}\left(1-{\left(1-\eta^{chan}\right)}^{n}\right)\right)\frac{\mu^{n}e^{-\mu }}{n!}f\left(\mu \right)d\mu \\ & & =\frac{1}{P_{S}}\int_{S}\left(e_{0}^{par}Y_{0}^{par}+e_{d}^{par}-e_{d}^{par}\sum_{n=0}^{\infty }\frac{{\left(\left(1-\eta^{chan}\right)\mu \right)}^{n}}{n!}\right)e^{-\mu }f\left(\mu \right)d\mu \\ & & =\frac{1}{P_{S}}\int_{S}\left(e_{0}^{par}Y_{0}^{par}+e_{d}^{par}-e_{d}^{par}e^{-\mu \eta^{chan}}\right)f\left(\mu \right)d\mu ,\\ & & E_{S}^{chan}=\frac{1}{P_{S}Q_{S}^{chan}}\int_{S}\left(e_{0}^{par}Y_{0}^{par}+e_{d}^{par}-e_{d}^{par}e^{-\mu \eta^{chan}}\right)f\left(\mu \right)d\mu . \end{array}$$

## Appendix E The derivation of Eqs. (23) and (24)

According to Eq. (17), we have

(65) $$\begin{array}{ccc} & & Q_{s}^{BA}=\sum_{n=0}^{\infty }P_{s}\left(n\right)Y_{n},\\ & & Q_{d1}^{BA}=\sum_{n=0}^{\infty }P_{d1}\left(n\right)Y_{n},\\ & & Q_{d2}^{BA}=\sum_{n=0}^{\infty }P_{d2}\left(n\right)Y_{n}, \end{array}$$

where s, d1, and d2 represent signalstate, decoystate1, and decoystate2, respectively.

When estimating $Y_{2}$, fluctuations in $Y_{1}$ can affect the solution of the linear programming problem, necessitating the elimination of $Y_{1}$. Taking $Q_{s}^{BA}$ and $Q_{d1}^{BA}$ as an example, we have

(66) $$\begin{array}{c} P_{d1}\left(1\right)Q_{s}^{BA}=\sum_{n=0}^{\infty }P_{d1}\left(1\right)P_{s}\left(n\right)Y_{n}, \end{array}$$

(67) $$\begin{array}{c} P_{s}\left(1\right)Q_{d1}^{BA}=\sum_{n=0}^{\infty }P_{s}\left(1\right)P_{d1}\left(n\right)Y_{n}. \end{array}$$

Subtracting Eq. (66) from Eq. (67), we have

(68) $$\begin{array}{ccc} P_{d1}\left(1\right)Q_{s}^{BA}-P_{s}\left(1\right)Q_{d1}^{BA} & = & \sum_{n=0}^{\infty }\left(P_{d1}\left(1\right)P_{s}\left(n\right)-P_{s}\left(1\right)P_{d1}\left(n\right)\right)Y_{n}\\ & = & \left(P_{d1}\left(1\right)P_{s}\left(0\right)-P_{s}\left(1\right)P_{d1}\left(0\right)\right)Y_{0}\\ & & +\;\sum_{n=2}^{\infty }\left(P_{d1}\left(1\right)P_{s}\left(n\right)-P_{s}\left(1\right)P_{d1}\left(n\right)\right)Y_{n}, \end{array}$$

where $P_{s}\left(n\right)$ and $P_{d1}\left(n\right)$ can be calculated with Eq. (12). However, it is impossible for us to calculate the case where n is infinite. Therefore, we need to set a truncation $n_{cut}$, and we have

(69) $$\begin{array}{ccc} P_{d1}\left(1\right)Q_{s}^{BA}-P_{s}\left(1\right)Q_{d1}^{BA} & = & \left(P_{d1}\left(1\right)P_{s}\left(0\right)-P_{s}\left(1\right)P_{d1}\left(0\right)\right)Y_{0}\\ & & +\;\sum_{n=2}^{n_{cut}}\left(P_{d1}\left(1\right)P_{s}\left(n\right)-P_{s}\left(1\right)P_{d1}\left(n\right)\right)Y_{n}\\ & & +\;\sum_{n=n_{cut}+1}^{\infty }\left(P_{d1}\left(1\right)P_{s}\left(n\right)-P_{s}\left(1\right)P_{d1}\left(n\right)\right)Y_{n} \end{array}$$

Next, we first need to prove that $P_{d1}\left(1\right)P_{s}\left(n\right)-P_{s}\left(1\right)P_{d1}\left(n\right)$ is greater than 0 for n≥2. According to Eq. (12), we have

(70) $$\begin{array}{ccc} P_{d1}\left(1\right)P_{s}\left(n\right)-P_{s}\left(1\right)P_{d1}\left(n\right) & = & \frac{1}{P_{d1}P_{s}}\int_{\mu_{v}}^{\mu_{d}}F\left(\mu \right)d\mu \int_{\mu_{d}}^{\mu_{s}}G\left(\mu \right)d\mu \\ & & -\;\frac{1}{P_{d1}P_{s}}\int_{\mu_{v}}^{\mu_{d}}G\left(\mu \right)d\mu \int_{\mu_{d}}^{\mu_{s}}F\left(\mu \right)d\mu \\ & = & \frac{1}{P_{d1}P_{s}}\int_{\mu_{v}}^{\mu_{d}}\int_{\mu_{d}}^{\mu_{s}}F\left(x\right)G\left(y\right)d\mu \\ & & -\;\frac{1}{P_{d1}P_{s}}\int_{\mu_{v}}^{\mu_{d}}\int_{\mu_{d}}^{\mu_{s}}F\left(y\right)G\left(x\right)d\mu \\ & = & \frac{1}{P_{d1}P_{s}}\int_{\mu_{v}}^{\mu_{d}}\int_{\mu_{d}}^{\mu_{s}}\left(F\left(x\right)G\left(y\right)-F\left(y\right)G\left(x\right)\right)d\mu ,\\ & & x\in \left(\mu_{v},\mu_{d}\right),y\in \left(\mu_{d},\mu_{s}\right) \end{array}$$

where

(71) $$\begin{array}{cc} P_{d1} & =\int_{\mu_{v}}^{\mu_{d}}f\left(\mu \right)d\mu , \end{array}$$

(72) $$\begin{array}{cc} P_{s} & =\int_{\mu_{d}}^{\mu_{s}}f\left(\mu \right)d\mu , \end{array}$$

(73) $$\begin{array}{cc} F(x) & =xe^{-x}f\left(x\right), \end{array}$$

(74) $$\begin{array}{cc} G(x) & =\frac{x^{n}e^{-x}}{n!}f\left(x\right). \end{array}$$

And f(x) is the probability density function shown in Eq. (16). Since

(75) $$\begin{array}{ccc} F(x)G(y)-F(y)G(x) & = & xe^{-x}f\left(x\right)\frac{y^{n}e^{-y}}{n!}f\left(y\right)-ye^{-y}f\left(y\right)\frac{x^{n}e^{-x}}{n!}f\left(x\right)\\ & = & \left(xy^{n}-yx^{n}\right)e^{-x-y}f\left(x\right)f\left(y\right)\frac{1}{n!}, \end{array}$$

we can determine the sign of F(x)G(y)−F(y)G(x) by checking the sign of $xy^{n}-yx^{n}$. Since y>x>0, $xy^{n}-yx^{n}$ is positive for n>1. In this way, we have

(76) $$\begin{array}{c} P_{d1}\left(1\right)P_{s}\left(n\right)-P_{s}\left(1\right)P_{d1}\left(n\right)\geq 0. \end{array}$$

And since $Y_{n}$ is less than 1, we have:

(77) $$\left(P_{d1}\left(1\right)P_{s}\left(n\right)-P_{s}\left(1\right)P_{d1}\left(n\right)\right)Y_{n}\geq 0,$$

(78) $$\left(P_{d1}\left(1\right)P_{s}\left(n\right)-P_{s}\left(1\right)P_{d1}\left(n\right)\right)Y_{n}\leq \left(P_{d1}\left(1\right)P_{s}\left(n\right)-P_{s}\left(1\right)P_{d1}\left(n\right)\right).$$

According to Eq. (69), we can obtain

(79) $$\begin{array}{ccc} P_{d1}\left(1\right)Q_{s}^{BA}-P_{s}\left(1\right)Q_{d1}^{BA} & \geq & \left(P_{d1}\left(1\right)P_{s}\left(0\right)-P_{s}\left(1\right)P_{d1}\left(0\right)\right)Y_{0}\\ & & +\;\sum_{n=2}^{n_{cut}}\left(P_{d1}\left(1\right)P_{s}\left(n\right)-P_{s}\left(1\right)P_{d1}\left(n\right)\right)Y_{n}, \end{array}$$

and

(80) $$\begin{array}{ccc} P_{d1}\left(1\right)Q_{s}^{BA}-P_{s}\left(1\right)Q_{d1}^{BA} & \leq & \left(P_{d1}\left(1\right)P_{s}\left(0\right)-P_{s}\left(1\right)P_{d1}\left(0\right)\right)Y_{0}\\ & & +\;\sum_{n=2}^{n_{cut}}\left(P_{d1}\left(1\right)P_{s}\left(n\right)-P_{s}\left(1\right)P_{d1}\left(n\right)\right)Y_{n}\\ & & +\;\sum_{n=n_{cut}+1}^{\infty }\left(P_{d1}\left(1\right)P_{s}\left(n\right)-P_{s}\left(1\right)P_{d1}\left(n\right)\right), \end{array}$$

where

(81) $$\begin{array}{ccc} & & \sum_{n=n_{cut}+1}^{\infty }\left(P_{d1}\left(1\right)P_{s}\left(n\right)-P_{s}\left(1\right)P_{d1}\left(n\right)\right)\\ & & \;=P_{d1}\left(1\right)\left(1-\sum_{n=0}^{n_{cut}}P_{s}\left(n\right)\right)-P_{s}\left(1\right)\left(1-\sum_{n=0}^{n_{cut}}P_{d1}\left(n\right)\right). \end{array}$$

According to Eqs. (79)–(81), we can obtain the linear programming in Eq. (23).

The derivation of Eq. (24) follows a similar process. First, according to Eq. (18), we have

(82) $$\begin{array}{ccc} & & Q_{s}^{BA}E_{s}^{BA}=\sum_{n=0}^{\infty }P_{s}\left(n\right)e_{n}Y_{n},\\ & & Q_{d1}^{BA}E_{d1}^{BA}=\sum_{n=0}^{\infty }P_{d1}\left(n\right)e_{n}Y_{n},\\ & & Q_{d2}^{BA}E_{d2}^{BA}=\sum_{n=0}^{\infty }P_{d2}\left(n\right)e_{n}Y_{n}. \end{array}$$

where s, d1, and d2 represent signalstate, decoystate1, and decoystate2, respectively.

Similarly, we first need to eliminate the term $e_{1}Y_{1}$. Taking signalstate (s) state and decoystate1 (d1) state as an example, we have

(83) $$P_{d1}\left(1\right)Q_{s}^{BA}E_{s}^{BA}=\sum_{n=0}^{\infty }P_{d1}\left(1\right)P_{s}\left(n\right)e_{n}Y_{n},$$

(84) $$P_{s}\left(1\right)Q_{d1}^{BA}E_{d1}^{BA}=\sum_{n=0}^{\infty }P_{s}\left(1\right)P_{d1}\left(n\right)e_{n}Y_{n}.$$

Then subtracting Eq. (83) from Eq. (84), we have

(85) $$\begin{array}{ccc} & & P_{d1}\left(1\right)Q_{s}^{BA}E_{s}^{BA}-P_{s}\left(1\right)Q_{d1}^{BA}E_{d1}^{BA}\\ & & \;=\sum_{n=0}^{\infty }\left(P_{d1}\left(1\right)P_{s}\left(n\right)-P_{s}\left(1\right)P_{d1}\left(n\right)\right)e_{n}Y_{n}\\ & & \;=\left(P_{d1}\left(1\right)P_{s}\left(0\right)-P_{s}\left(1\right)P_{d1}\left(0\right)\right)e_{0}Y_{0}\\ & & \;+\;\sum_{n=2}^{n_{cut}}\left(P_{d1}\left(1\right)P_{s}\left(n\right)-P_{s}\left(1\right)P_{d1}\left(n\right)\right)e_{n}Y_{n}\\ & & \;+\;\sum_{n=n_{cut}+1}^{\infty }\left(P_{d1}\left(1\right)P_{s}\left(n\right)-P_{s}\left(1\right)P_{d1}\left(n\right)\right)e_{n}Y_{n} \end{array}$$

According to Eq. (76), and $e_{n}Y_{n}$ is less than 1, we have

(86) $$\left(P_{d1}\left(1\right)P_{s}\left(n\right)-P_{s}\left(1\right)P_{d1}\left(n\right)\right)e_{n}Y_{n}\geq 0,$$

(87) $$\left(P_{d1}\left(1\right)P_{s}\left(n\right)-P_{s}\left(1\right)P_{d1}\left(n\right)\right)e_{n}Y_{n}\leq \left(P_{d1}\left(1\right)P_{s}\left(n\right)-P_{s}\left(1\right)P_{d1}\left(n\right)\right).$$

Combining it with Eq. (85), we can obtain

(88) $$\begin{array}{ccc} & & P_{d1}\left(1\right)Q_{s}^{BA}E_{s}^{BA}-P_{s}\left(1\right)Q_{d1}^{BA}E_{d1}^{BA}\\ & & \;\geq \left(P_{d1}\left(1\right)P_{s}\left(0\right)-P_{s}\left(1\right)P_{d1}\left(0\right)\right)e_{0}Y_{0}\\ & & \;+\;\sum_{n=2}^{n_{cut}}\left(P_{d1}\left(1\right)P_{s}\left(n\right)-P_{s}\left(1\right)P_{d1}\left(n\right)\right)e_{n}Y_{n}, \end{array}$$

and

(89) $$\begin{array}{ccc} & & P_{d1}\left(1\right)Q_{s}^{BA}E_{s}^{BA}-P_{s}\left(1\right)Q_{d1}^{BA}E_{d1}^{BA}\\ & & \;\leq \left(P_{d1}\left(1\right)P_{s}\left(0\right)-P_{s}\left(1\right)P_{d1}\left(0\right)\right)e_{0}Y_{0}\\ & & \;+\sum_{n=2}^{n_{cut}}\left(P_{d1}\left(1\right)P_{s}\left(n\right)-P_{s}\left(1\right)P_{d1}\left(n\right)\right)e_{n}Y_{n}\\ & & \;+\sum_{n=n_{cut}+1}^{\infty }\left(P_{d1}\left(1\right)P_{s}\left(n\right)-P_{s}\left(1\right)P_{d1}\left(n\right)\right), \end{array}$$

where

(90) $$\begin{array}{ccc} & & \sum_{n=n_{cut}+1}^{\infty }\left(P_{d1}\left(1\right)P_{s}\left(n\right)-P_{s}\left(1\right)P_{d1}\left(n\right)\right)\\ & & \;=P_{d1}\left(1\right)\left(1-\sum_{n=0}^{n_{cut}}P_{s}\left(n\right)\right)-P_{s}\left(1\right)\left(1-\sum_{n=0}^{n_{cut}}P_{d1}\left(n\right)\right). \end{array}$$

According to Eqs. (88)–(90), we can obtain the linear programming in Eq. (24).

The method is also applicable to other light sources, such as weak coherent light sources, provided that they satisfy Eq. (76) for n≥2. In fact, Eq. (76) generally holds because it can be interpreted as: when the light intensity is high, the proportion of multi-photon (n-photon) states increases more rapidly than that of single-photon states.
